# Stage-Specific Proteomic Signatures of Matrix Vesicles Support Functional Transitions during Biomineralization

**DOI:** 10.1021/acs.jproteome.6c00508

**Published:** 2026-10-20

**Authors:** Juçara Gastaldi Cominal, Larwsk Hayann, Maryanne Trafani de Melo, Iara Souza Lima, Angie Lissette Atoche Puelles, Lucas Fabricio Bahia Nogueira, Saida Mebarek, Ana Paula Ramos, José Luis Millán, Massimo Bottini, Pietro Ciancaglini

**Affiliations:** † Departamento de Química, Faculdade de Filosofia, Ciências e Letras de Ribeirão Preto, Universidade de São Paulo, 14040-901 Ribeirão Preto, Brasil; ‡ Department of Experimental Medicine, 9318 University of Rome Tor Vergata, 00133 Rome, Italy; § Université de Lyon1, UMR CNRS 5246, 69622 Villeurbanne, France; ∥ Sanford Children’s Health Research Center, 4355 Sanford Burnham Prebys Medical Discovery Institute, La Jolla, La Jolla, California 92037, United States

**Keywords:** matrix vesicle, osteoblast, osteogenesis, proteomic, biomineralization, mineral propagation

## Abstract

Matrix vesicles (MVs) are extracellular vesicles released by mineralizing-competent cells, including chondrocytes and osteoblasts. Although MVs are classically associated with biomineralization, whether distinct MV populations are produced throughout osteoblast differentiation remains poorly understood. Here, we investigated the biophysical, biochemical, and proteomic profiles of MVs isolated throughout osteogenic differentiation to evaluate the hypothesis of functional transitions during biomineralization progression. MVs were isolated from osteoblast cultures at three time points, representing the onset, establishment, and propagation stages of biomineralization. Biophysical and biochemical characterization revealed temporal differences in MV properties. Stage-specific proteomic signatures were identified among MV groups. Early stage MVs (7–14 days) exhibited enrichment of proteins associated with the extracellular matrix (ECM) organization, intracellular trafficking, cytoskeletal dynamics, and adhesion, suggesting a role in preparing the mineralizing microenvironment. In contrast, later-stage MVs (14–21 days) showed enrichment of proteins related to ECM interactions, calcium response, proteoglycan binding, metabolism, and regulatory signaling pathways, consistent with enhanced mineral propagation. Collectively, our findings demonstrate that distinct MVs are produced throughout osteoblast differentiation and support a functional transition model during biomineralization, in which MV cargo and properties are dynamically coordinated with the temporal demands of ECM mineralization.

## Graphical abstract

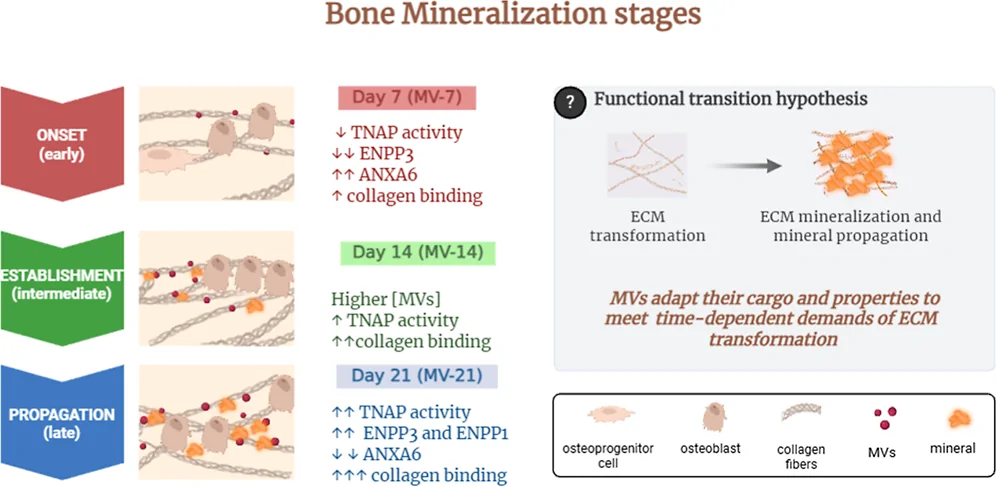

## Introduction

Endochondral and intramembranous ossification are well-coordinated processes that occur throughout our lifespan and are necessary for the formation, modeling, and remodeling of the skeleton to adapt to growth, meet mechanostatic demands, promote tissue repair, and to keep calcium–phosphate homeostasis and endocrine functions.^1,2^ Osteoblasts play a key role acting as bone-forming agents, and part of their function is modulated by the production and release of extracellular vesicles (EVs). EVs are nonreplicating particles ranging in size from several tens to a few hundred nanometers, composed of a lipid bilayer, that all cells release into the surrounding environment. Their main function is to mediate intercellular communication, locally or over distances, transporting specific cargoes, such as proteins, lipids, and nucleic acids, both within their lumen and on their membranes.^3–5^ Matrix vesicles (MVs) are a special class of EVs that are found entrapped (chemically or physically) into the extracellular matrix (ECM). The current model describes MVs as acting locally to modulate calcium–phosphate homeostasis and mineral formation due to their enrichment in key mineralization-associated enzymes and proteins, including tissue-nonspecific alkaline phosphatase (TNAP), orphan phosphatase 1 (PHOSPHO1), and the ectonucleotide pyrophosphatases/phosphodiesterases (ENPP).^6–8^ The mechanism underlying MVs biogenesis remains unclear, with both a mechanism based on plasma membrane blebbing and a mechanism based on the endosomal pathway being postulated.^9,10^ More recently, a hybrid biogenesis hypothesis has been proposed with the involvement of different models, including a lipid-centered mechanism.^11^ Taking into consideration that cells simultaneously produce different types of EVs using different pathways, and different types of EVs could share similar functions, it is a challenge to isolate a pure EVs population as well as to determine their precise biogenesis mechanism.

MVs and EVs recovered from conditioned medium (media-EVs, commonly termed exosomes) represent distinct EVs populations that share several characteristics, including size, morphology, and subsets of proteins and lipids. However, their functional distinction is based not solely on their association with the ECM and properties relevant to biomineralization, such as calcium and phosphate enrichment, elevated TNAP activity, collagen/ECM binding, and mineralization capacity.^3,9,12^ In addition, media-EVs have been implicated in osteoblast differentiation, primarily through cell-to-cell communication and the regulation of cellular functions, whereas the initiation of biomineralization is particularly associated with MVs.^3^

Although previous studies have suggested a role for MVs in the early stages of mineral nucleation and propagation into the ECM, it is unclear whether the properties of MVs change throughout the mineralization process. Considering the dynamics and complexity of bone formation and remodeling processes, the temporal changes in the extracellular matrix and resident cells, and the fact that the content of EVs reflects the state of the cells of origin,^13^ we evaluated MVs isolated during the biomineralization process and explored their major characteristics, functions, and behavior. To this end, we used a well-established and reliable preosteoblast cell line model (MC3T3-E1), which is known to exhibit three well-characterized stages: up to 7 days (early differentiation stage), 14 days (high alkaline phosphatase activity, elevated collagen secretion, and high MV-derived yield), and 21 days (full mineralization of the extracellular matrix). Our goal is to better understand MVs production and its involvement in mineral formation and propagation.

## Materials and Methods

### Osteoblast Culture and MVs Isolation

The MC3T3-E1 subclone 14 mouse preosteoblast cell line (CRL-2594, ATCC) was initially cultured and expanded in base medium, Minimum Essential Medium (α-MEM, Gibco/Thermo Fisher Scientific), supplemented with 10% fetal bovine serum (Gibco/Thermo Fisher Scientific) and 1% penicillin–streptomycin (10.000 U/mL, Gibco/Thermo Fisher Scientific) at 37 °C and 5% CO_2_. Cells tested negative for mycoplasma contamination. Osteogenesis was induced by supplementing base medium with ascorbic acid (50 μg/mL, Sigma-Aldrich) and β-glycerophosphate (10 mM, Sigma-Aldrich). The medium was renewed every 48 h, and culture was kept for 7, 14, and 21 days in T175 cm^2^ flasks.

MVs were isolated from the ECM following an adapted protocol of Buchet et al.^11^ (Figure 1A). Briefly, cells and the ECM were washed twice with PBS buffer and incubated with digestion solution for 1 h at 37 °C with low agitation. Samples were subjected to consecutive centrifugations at 4 °C: first at 600*g* for 20 min, second at 20,000*g* for 20 min, and third at 80,000*g* for 60 min. After each step, the supernatant was transferred to a new tube. MVs were resuspended in SCL buffer and stored at −20 °C. Digestion solution was composed of SCL buffer containing CaCl_2_ (2 mmol L^–1^, Sigma-Aldrich) and Type I Collagenase from *Clostridium histolyticum* (200 U/mL, Sigma-Aldrich). The SCL buffer (pH 7.6) was composed of d-glucose (5.55 mmol L^–1^), KCl (12.7 mmol L^–1^), MgCl_2_ (0.57 mmol L^–1^), NaCl (100 mmol L^–1^), NaHCO_3_ (1.83 mmol L^–1^), Na_2_SO_4_ (0.7 mmol L^–1^), sucrose (63.5 mmol L^–1^), and tris (16.5 mmol L^–1^, 2-amino-2-hydroxymethylpropane-1,3-diol).

**Figure 1:**
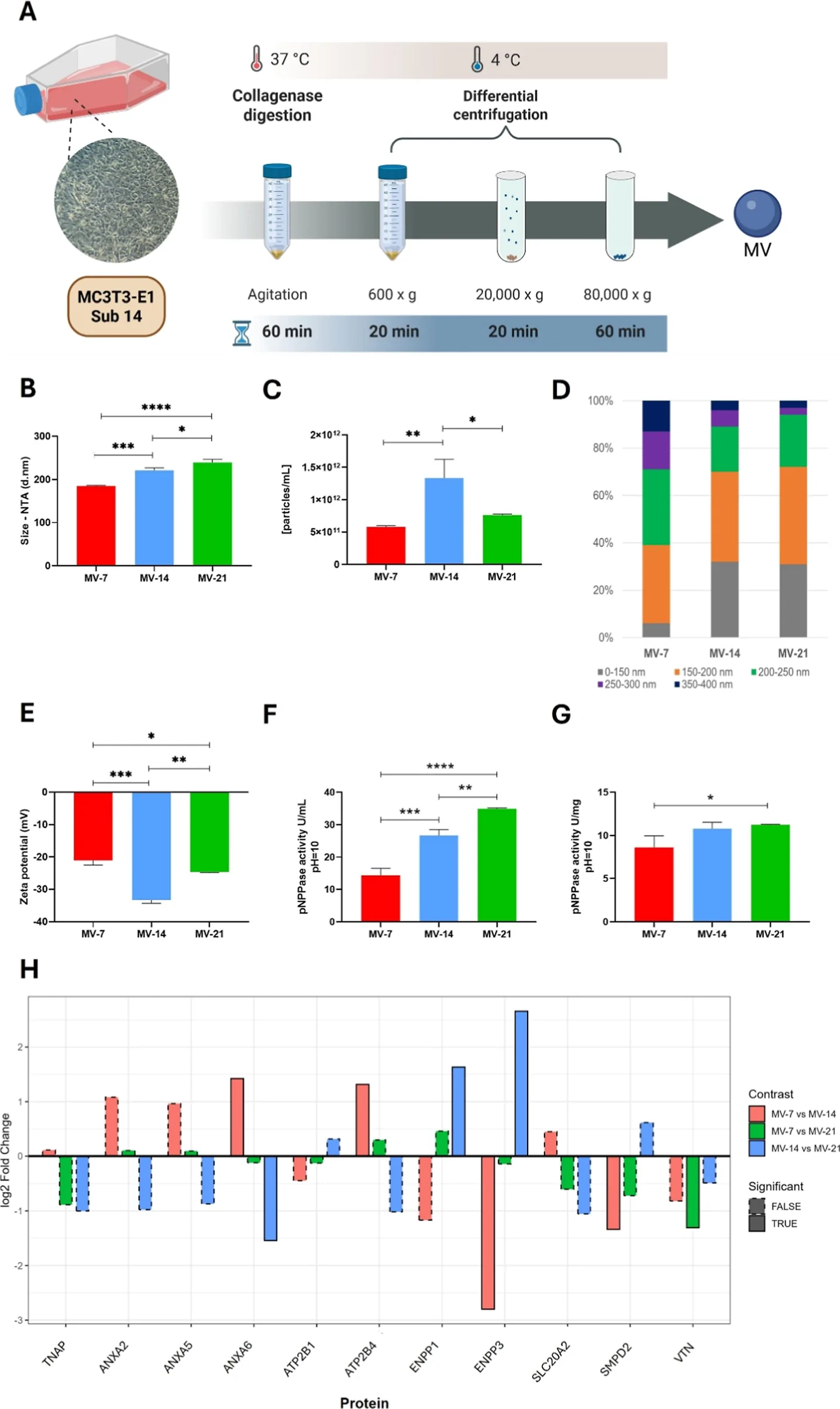
Physical and biochemical characterization of matrix vesicles (MVs). (A) Scheme of the MVs isolation protocol. (B–G) Biophysical and biochemical properties of MVs are isolated at 7 days (MV-7), 14 days (MV-14), and 21 days (MV-21) of cell culture. Analyses were performed in triplicate, and plotted values represent the mean (bars) ± standard deviation. Statistical analysis was conducted using two-way ANOVA, followed by Tukey’s posthoc test for multiple comparisons. Differences between groups were considered statistically significant at *p* < 0.05. Significant levels: *p* ≤ 0.05 (*), *p* ≤ 0.01 (**), *p* ≤ 0.001 (***), *p* ≤ 0.0001 (****). (B–D) Nanoparticle tracking analysis (NTA). (B) Size. (C) Particle concentration per mL. (D) Size distribution. (E) Zeta potential. (F,G) Tissue-nonspecific alkaline phosphatase (TNAP) activity measured at pH 10 using *p*-nitrophenyl phosphate (*p*NPP) as a substrate. (H) Abundance of biomineralization-associated proteins across groups. Statistical analysis was performed using the limma package. Differences were considered significant at *p* < 0.05 (TRUE), while nonsignificant results are indicated as FALSE.

### Nanoparticle Tracking Analysis, Dynamic Light Scattering Characterization, and Protein Quantification

MVs were diluted 1:1.000 in water, and their size and concentration were determined by nanoparticle tracking analysis (NS3000, Malvern). Data acquisition used five video files of 60 s. The zeta potential of MVs was determined using dynamic light scattering (Malvern). For this analysis, MVs were diluted 1:2000 in water, and data were acquired in triplicate. Total protein content was quantified using the Bradford protein assay (Bio-Rad) according to the manufacturer’s instructions.

### Atomic Force Microscopy

MVs were diluted 1:200 in water, filtered through a sterile 0.22 μm filter (PVDV Durapore Membrane, Millipore), and fixed with glutaraldehyde (2% w/v). Morphological analyses by AFM were performed in a SPM-9600 scanning probe microscope (Shimadzu Co.) operating in intermittent mode.

### Mineralization Assay

MVs (20 μg of total protein) were incubated in a 96-well plate for 18 h at 37 °C, in a solution containing SCL buffer, 2 mmol L^–1^ CaCl_2_, and 2.0 mmol L^–1^ ATP. The turbidity was measured at 340 nm, with 5 min intervals, in triplicate.^14^ After the incubation, the supernatant was removed and each well was washed twice with ultrapure water (Direct-Q 5, Merck Millipore). The 96-well plate was placed in a dissector with silica and allowed to dry for 1 week. The produced mineral was analyzed by means of three independent attenuated total reflectance–Fourier transform infrared spectroscopy (ATR-FTIR) readings (IRPrestige-21, Shimadzu Co.) using a ZnSe crystal attenuated total reflectance accessory.

### Preparation of Collagen Scaffold

Collagen scaffold was prepared using a cylindrical Teflon template filled with a solution of Type I collagen (∼10 mg·mL^–1^) extracted and purified from mice tails, as previously described.^15^ The template was then exposed to an NH_3_(g) atmosphere and dehydrated in a climate chamber at 37 °C with 40% of humidity.

### Collagen Binding Assay

In a 96-well plate, MVs (20 μg of total protein) were placed onto the collagen scaffold with SCL buffer containing 2.0 mmol L^–1^ of CaCl_2_ and 2.0 mmol L^–1^ of adenosine triphosphate (ATP) and incubated at 37 °C and 5% of CO_2_. The buffer was removed from each well, and all scaffolds were fixed using 4% paraformaldehyde (w/v) for 30 min and 1% OsO_4_ (w/v) for 2 h. Subsequently, they were rinsed and treated with thiocarbohydrazide, followed by ethanol dehydration and critical point-drying with liquid CO_2_. The dried samples were then mounted on aluminum stubs, gold-coated, and analyzed using scanning electron microscopy (SEM; Shimadzu SS-500 microscope, Shimadzu Co.). Morphological changes in collagen-based scaffolds were examined after 24 h incubation.

### Proteomic Analysis of MVs

Tandem mass tag (TMT) quantitative proteomics analysis was performed using three biological replicates and 10 μg of total protein of each sample. Sample digestion and labeling followed the described protocol.^16^ Peptides were fractionated under high pH conditions,^17^ yielding 6 fractions.

After sample preparation, the liquid chromatography–tandem mass spectrometry (LC–MS/MS) analysis was performed. Briefly, peptides were separated using an Ultimate 3000 nano RSLC system (Dionex) equipped with a trapping cartridge (Precolumn C18 PepMap100, 5 mm, 300 μm i.d., 5 μm, 100 Å) and an analytical column (Acclaim PepMap 100. 75 × 50 cm C18, 3 mm, 100 Å) connected to a nanospray-Flex ion source. The peptides were loaded onto the trap column at 30 μL min^–1^ using solvent A (0.1% formic acid, v/v) and eluted using a gradient from 2% to 40% solvent B (0.1% formic acid in acetonitrile, v/v) over 2 h at 0.3 μL min^–1^ (all solvents were of LC–MS grade). The Orbitrap Fusion Lumos was operated in positive ion mode with a spray voltage of 2.4 kV and capillary temperature of 275 °C. Full scan MS spectra with a mass range of 375–1500 m z^–1^ were acquired in profile mode using a resolution of 120,000 [maximum fill time of 50 ms or a maximum of 4 × 10^5^ ions (AGC)] and an RF lens setting of 30%. Fragmentation was triggered for 3 s cycle time for peptide-like features with charge states of 2–7 on the MS scan (data-dependent acquisition). Precursors were isolated using the quadrupole with a window of 0.7 m z^–1^ and fragmented with a normalized collision energy of 38. Fragment mass spectra were acquired in profile mode with a resolution of 30,000. Maximum fill time was set to 64 ms or an AGC target of 1 × 10^5^ ions. The dynamic exclusion was set to 45 s. Acquired data were analyzed using FragPipe^18^ against a reverse UniProt FASTA *Mus musculus* database (UP000000589), including common contaminants. The following modifications were considered: carbamidomethyl (C, fixed), TMT10plex (K, fixed), acetyl (N-term, variable), oxidation (M, variable), and TMT10plex (N-term, variable). The mass error tolerance for full scan MS spectra was set to 10 ppm and for MS/MS spectra to 0.02 Da. A maximum of 2 missed cleavages were allowed. A minimum of 2 unique peptides with a peptide length of at least seven amino acids and a false discovery rate (FDR) below 0.01 were required on the peptide and protein level.^19^ Data analysis was performed following the steps described below. The raw output files of FragPipe [protein.tsv—files^20^ were processed using the R programming language (ISBN 3-900051-07-0). Log_2_ transformed raw TMT reporter ion intensities were first cleaned for batch effects using the “removeBatchEffects” function of the limma package^21^ and further normalized using the variance stabilization normalization package.^22^ Proteins were tested for differential expression using a moderated *t*-test by applying the limma package. The replicate information was added as a factor in the design matrix given as an argument to the “lmFit” function of limma. For the purposes of this study, proteins were considered differentially expressed if they exhibited an FDR < 5% and a fold change (FC) ≥ 2 (|log_2_ FC| ≥ 1).

### Statistical Analysis

For analyses other than proteomic data, groups were compared using two-way ANOVA, followed by Tukey’s post hoc test. Statistical significance was set at *p* < 0.05, and results were presented graphically as bar graphs or scatter plots, as appropriate. Data are reported as mean ± standard deviation (SD). All statistical analyses were performed using GraphPad Prism 8 (GraphPad Software). Statistical analysis of proteomic data was performed separately in the R environment (v4.5.1) using Rstudio (v2025, 09, 2). In summary, log_2_-transformed TMT-reported ion intensities were corrected for batch effects and normalized using variance stabilization normalization, followed by differential expression, as described before.

## Results

### MVs Exhibit Time-Dependent Biophysical and Biochemical Characteristics during Biomineralization

MVs were isolated at three different time-points of osteoblast differentiation: at day 7 (MV-7), day 14 (MV-14), and day 21 (MV-21), representing the onset of biomineralization (early differentiation and ECM remodeling), the establishment of a mineralization-prone matrix, and the propagation of mineralization within the ECM, respectively. Figure 1A illustrates the isolation process.

Significant temporal changes were observed in MVs (summarized in Table 1 and Figure 1B–G). The biophysical and biochemical properties of MV-7 were different from those of MV-14 and MV-21. MV-7 was smaller (median size of 185 nm) with a zeta potential of −21 mV and displayed the lowest concentration of particles (5.81 × 10^11^ particles/mL) (Figure 1B,C,E). The concentrations of MV-14 and MV-21 were 101% and 31%, respectively, greater than that of MV-7. Importantly, MV-14 showed not only the highest concentration of particles but also the lowest zeta potential among the samples analyzed (Figure 1E). Size distribution analysis showed that MV-14 and MV-21 had a similar size distribution, with particles sized between 150 and 200 nm accounting for 38.3% and 40.7%, respectively (Figure 1D). The MV-7 group showed two abundant populations: 32.9% of particles sized between 150 and 200 nm and 32.1% of particles sized between 200 and 250 nm. Interestingly, small particles (<150 nm) constituted the second largest population in MV-14 (31.3%) and MV-21 (31.1%), while the smallest in MV-7 (5.4%) (Figure 1D). TNAP-specific activity progressively increased over time, with the highest levels detected on MV-21 (Figure 1F,G).

**Table 1:** Biochemical and Biophysical Characteristics of MVs^a^

parameters	MV-7	MV-14	MV-21
NTA			
Concentration (particles/mL)	5.81 × 10^11^	1.33 × 10^12^	7.63 × 10^11^
SE	1.1 × 10^10^	1.6 × 10^11^	8.7 × 10^9^
SD	1.9 × 10^10^	2.9 × 10^11^	1.5 × 10^10^
Size (nm)	185	221	239
SE	0.9	3.4	4.2
SD	1.6	5.9	7.3
zeta potential (mV)	-21	-33.3	-24.6
total protein (μg/μL)	1.6	2.5	3.1
TNAP activity (pH=10)			
U/mg	8.6	10.8	11.2
U/mL	14.3	26.7	34.8
AFM			
Volume (nm)^3^	394335	106941	425844
SD	1044023	185515	1210247
Z (nm)	10.32	6.85	21.29
SD	3.4	1.1	2.6
Roughness	0.66	0.584	0.786
SD	0.3	0.4	0.3
n	301	224	184

a Atomic force microscopy (AFM); matrix vesicles (MVs); nanoparticle tracking analysis (NTA); standard deviation (SD); standard error (SE); tissue-nonspecific alkaline phosphatase (TNAP). Values were reported as mean ± SE.

A quick look at the proteomic analysis revealed that the abundance of key proteins associated with biomineralization did not show significant variations among groups. Among these proteins are annexins (ANXA2 and ANXA5), TNAP, vitronectin (VTN), plasma membrane calcium transport ATPase 1 (ATP2B1), and sodium-dependent phosphate transporter 2 (SLC20A2/PIT2). In contrast, annexin A6 (ANXA6), ENPP1, ENPP3, plasma membrane calcium transport ATPase (ATP2B4), and neutral sphingomyelinase (SMPD2) showed differential abundance between groups (Figure 1H). Furthermore, the expression of EV-associated markers was studied in all groups in accordance with MISEV 2023 (Minimal Information for Studies of Extracellular Vesicles) guidelines.^23^ Markers of both plasma-membrane-derived vesicles [integrin beta 1 (ITGB1) and 72 kDa type IV collagenase (MMP2)] and those derived from an endolysosomal pathway [tumor susceptibility gene 101 protein (TSG101), vacuolar protein-sorting-associated protein 36 (VPS36), and lysosome-associated membrane glycoprotein 1 (LAMP1)] were identified. Furthermore, the expression of canonical EV-associated tetraspanins (CD81, CD9, and CD63) was unchanged among the different groups.

Morphological characterization of MVs by means of AFM revealed round nanostructures of similar size but with distinct surface and height features (Figure 2). MV-7 exhibited a moderate surface roughness and an average height of approximately 17 nm. Phase images of MV-7 showed homogeneous and stiffer contours, with heterogeneous and softer core. MV-14 displayed a more defined spherical morphology, decreased roughness, and an increased height (around 24 nm). Its contours were homogeneous and stiff, like MV-7; however, the core was more heterogeneous than that of MV-7, exhibiting both softer and stiffer regions, suggesting the coexistence of domains with distinct mechanical and/or molecular properties. MV-21 presented a continuous surface with the highest roughness and pronounced curvature, reaching heights of approximately 34 nm. Phase images showed MV-21 with a heterogeneous core and uniformly stiff contours, except for the presence of a small peripheral protuberance. The numerical values of AFM analysis can be found in Table 1.

**Figure 2:**
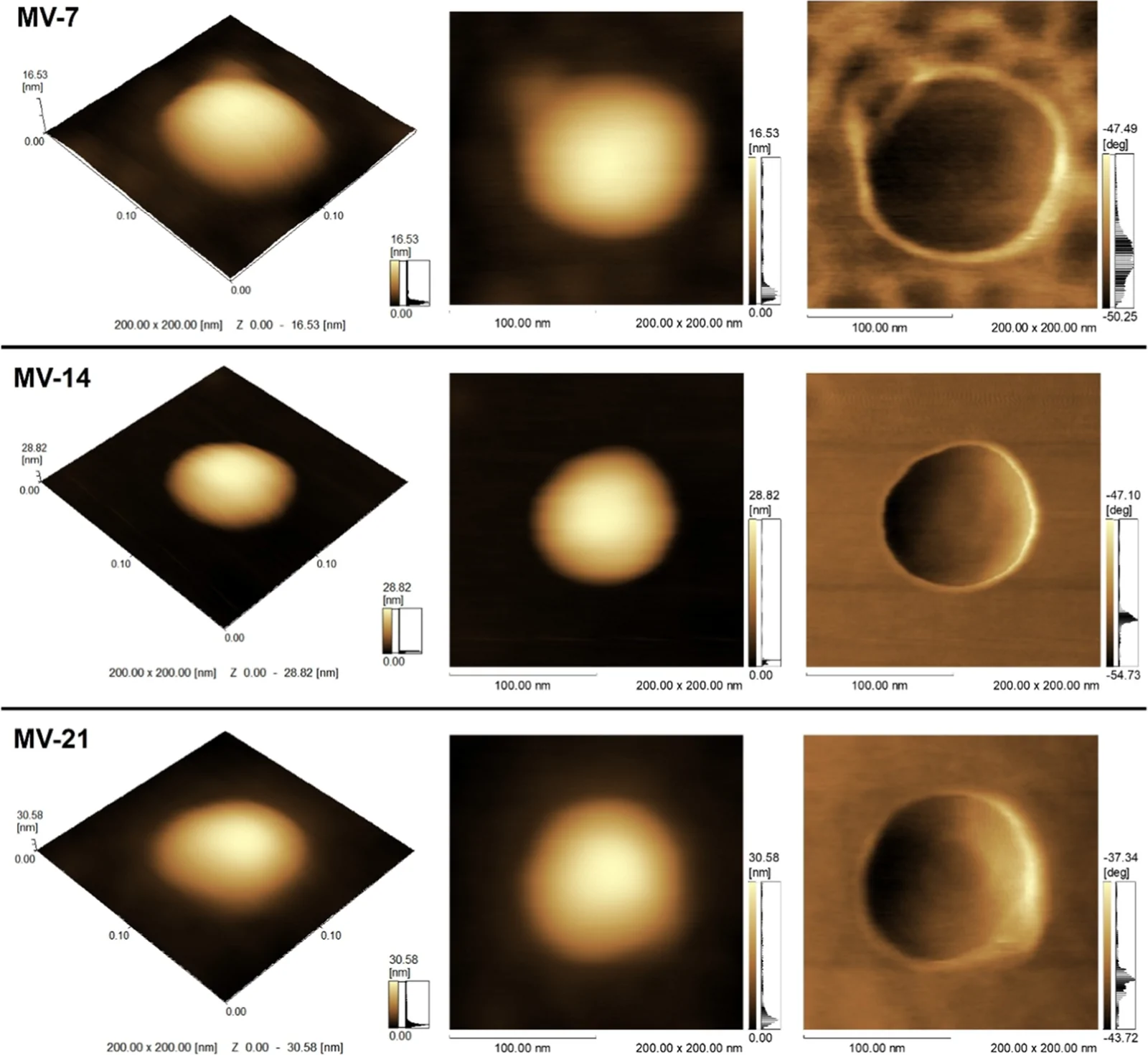
Morphological characterization of MVs by atomic force microscopy (AFM). Representative AFM images of topography (height) three-dimensional (right) and phase-contrast micrographs. Images were acquired using a Shimadzu SPM-9600 scanning probe microscope, operating in a tap mode. For each sample, at least 100 vesicles were analyzed. MVs were grouped according to the day of isolation: day 7 (MV-7), day 14 (MV-14), and day 21 (MV-21).

Next, an in vitro mineralization assay was performed (Figure 3A) to evaluate the mineralization potential of each group of MVs. The turbidity assay demonstrated that all MVs can mineralize, with MV-21 showing the highest activity, followed by MV-14 and MV-7. In particular, the onset of mineralization was delayed in MV-7 and MV-14 compared with MV-21. Chemical characterization of the products generated during the turbidity test was performed by ATR-FTIR (Figure 3B), revealing products with similar chemical compositions, consistent with biological apatite precursors.

**Figure 3:**
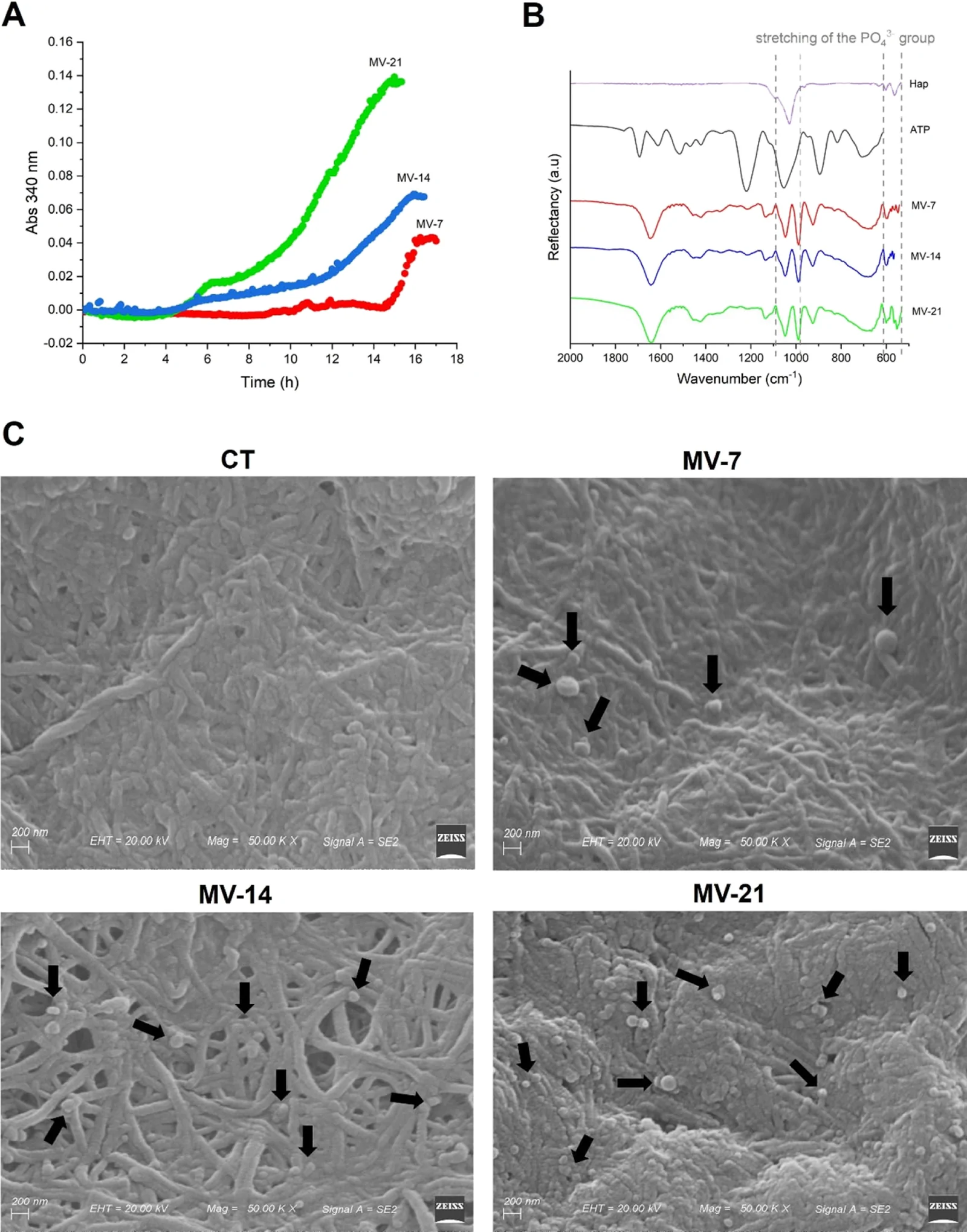
In vitro mineralization potential of matrix vesicles (MVs). MVs were grouped according to the day of isolation: day 7 (MV-7, red), day 14 (MV-14, blue), and day 21 (MV-21, green). Data represent the mean of triplicate experiments. (A) Turbity assay performed in the presence of adenosine triphosphate (ATP) and calcium. (B) Attenuated total reflectance**–**Fourier transform infrared spectroscopy (ATR-FTIR) spectra. ATP and hydroxyapatite (HAp) standards were used for comparison. Phosphate (PO_4_^3–^) stretching vibrations are indicated by dashed lines. (C) Scanning electron microscopy images of the collagen anchoring assay. MVs were incubated within a three-dimensional scaffold composed of type I collagen. Micrographs were obtained at 50,000× magnification, and the white scale bar represents 200 nm. Black arrows indicate the interaction of the vesicles and scaffolds.

The collagen-anchoring capacity of MVs was assessed using a 3D collagen scaffold (Figure 3C). All MVs groups retained the ability to interact with type I collagen, which is the predominant organic component of the bone matrix. However, MV-14 and MV-21, derived from intermediate and late stages of biomineralization, showed greater collagen anchoring than MV-7, which represents an early stage.

### Proteomic Remodeling Reflects a Shift from Cellular Support to Extracellular Execution

Proteomic analysis of MVs identified 1605 gene products. Principal component analysis (PCA; Figure 4A) revealed temporal clustering separated into two main groups. The biplot integrates the projection of samples with vectors representing proteins with the highest loadings, thereby highlighting the variables that contribute the most to the observed separation. The first principal component (PC1) explained approximately 47.5% of the variance, separating MV-7 from MV-14 and MV-21, indicating a marked shift in the MV proteomic profile after day 7. The second principal component (PC2), accounting for approximately 39.5% of the variance, further separated MV-14 and MV-21, revealing differences not captured by PC1, suggesting more subtle maturation-related changes occurring after day 14.

**Figure 4:**
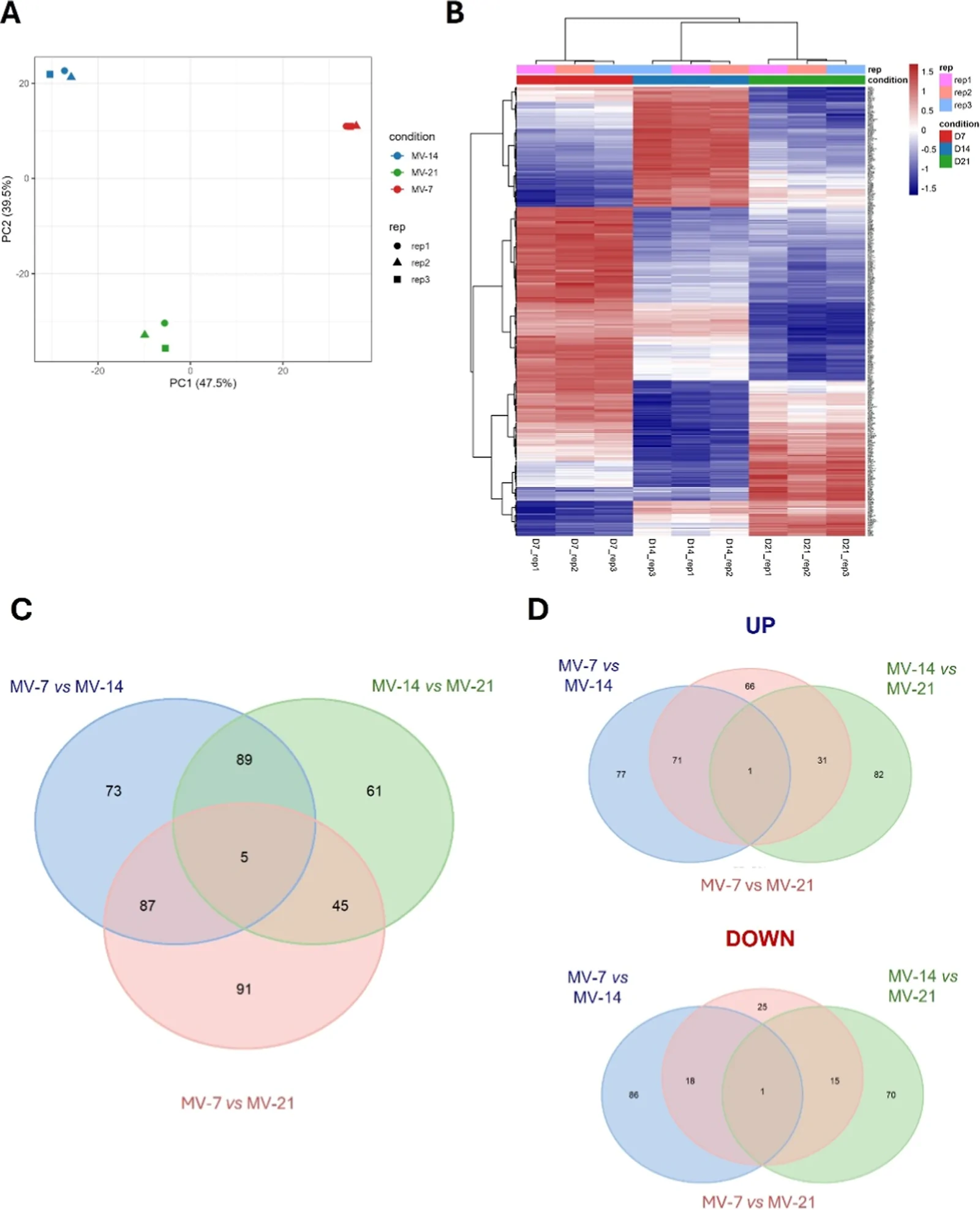
Time shifts of MVs proteomics. (A) Principal component analysis (PCA) indicated two clusters (PC1 and PC2). MVs were grouped according to the day of isolation: day 7 (MV-7, red), day 14 (MV-14, blue), and day 21 (MV-21, green). Experimental replicates (triangle, circle, and square; Var, variance). (B) Heatmap of differentially abundant proteins (*n* = 451). Each group was analyzed in triplicate (columns; rep). From left to right, the samples are MV-7, MV-14, and MV-21. (C) Venn diagram illustrating the overlapping (intersections) and unique relationships among proteins identified in the pairwise comparisons between MVs groups. (D) Venn diagrams showing upregulated (top) and downregulated (bottom) proteins across comparisons. Proteins included in both the heatmap and Venn diagrams met the following criteria: false discovery rate (FDR) < 0.05 and |log_2_(fold change)| ≥ 1.

Differential protein expression analysis (FDR < 0.05, FC ≥ 2; |log_2_ FC| ≥ 1) identified 451 proteins with significant changes, revealing distinct proteomic profiles across all pairwise comparisons. A global heatmap was generated using the combined set of differentially expressed proteins (Figure 4B), highlighting the overall differences in protein abundance and content.

The Venn diagram (Figure 4C­(Figure 1C, Supporting Information 1A) highlighted that most differentially expressed proteins were comparison-specific, with 73 proteins unique to MV-7 vs MV-14, 61 proteins unique to MV-14 vs MV-21, and 91 proteins unique to MV-7 vs MV-21. Notably, only five proteins were common to all three comparisons, indicating a largely stage-specific remodeling of the MVs proteome content during osteogenic differentiation. The shared proteins included chondroitin sulfate *N*-acetylgalactosaminyltransferase 1 (CSGALNACT1), H2B clustered histone 14 (H2BC14), pleiotrophin (PTN), poliovirus receptor (PVR), and SET nuclear proto-oncogene (SET) (Figure 4C).

Directional Venn diagrams were generated separately for upregulated and downregulated proteins, enabling the identification of activation and repression signatures across the course of the biomineralization process (Figure 4D, Supporting Information 1B).

Gene ontology (GO) enrichment analysis was subsequently performed on the sets of unique proteins identified in each comparison, revealing significant terms across biological process (BP), cellular compartment (CC), and molecular function (MF) (Supporting Information 2) categories.

MV-7 vs MV-14 comparison showed enrichment BP terms connected to epidermis development, muscle system contraction, and amide metabolic process. In the CC category, enrichment was observed for terms related to cytoskeletal and contractile structures, such as contractile muscle fiber, stress fiber, actin filament bundle, myofibril, and sarcomere. MF revealed enrichment of protein binding-related terms, including collagen binding, carbohydrate binding, actin binding, and calmodulin binding.

MV-14 vs MV-21 comparison revealed, within the BP category, the most enriched terms included response to calcium ion, response to metal ion, fatty acid metabolic process, amide metabolic process, and pathways related to cell–matrix and cell–substrate adhesion, as well as extracellular matrix organization and external encapsulating structure organization. Terms associated with signaling regulation, such as the extracellular signal-regulated kinase cascade (ERK1 and ERK2), were also represented. For CC, the terms predominantly present were linked to membrane and cellular compartments, including collagen-containing extracellular matrix, basement membrane, basal part of the cell, apical plasma membrane, membrane raft, and transport vesicle. In the MF category, enrichment was observed for activities related to matrix interaction and adhesion, including proteoglycan binding, extracellular matrix structural constituents, integrin binding, cell adhesion molecule binding, and glycosaminoglycan binding. Additional terms, such as metallopeptidase activity and peptidase regulator activity, were also identified.

Regarding MV-7 vs MV-21 comparison, the BP category identified terms associated with protein synthesis and processing, including cytoplasmatic translation, ribosome biogenesis, protein folding, and protein stabilization. For CC, enrichment was linked to ribosome-associated compartments, followed by microtubule and synapse-related components. In the MF category, the most represented terms included structural constituent of ribosome, ATP-dependent protein folding chaperone, unfolded protein binding, and mRNA binding. Binding activities related to ubiquitin ligases, actin filaments, and cell-adhesion molecules were also detected.

The top five enriched GO terms were represented as a network plot (Figure 5), highlighting the relationships among the associated proteins.

**Figure 5:**
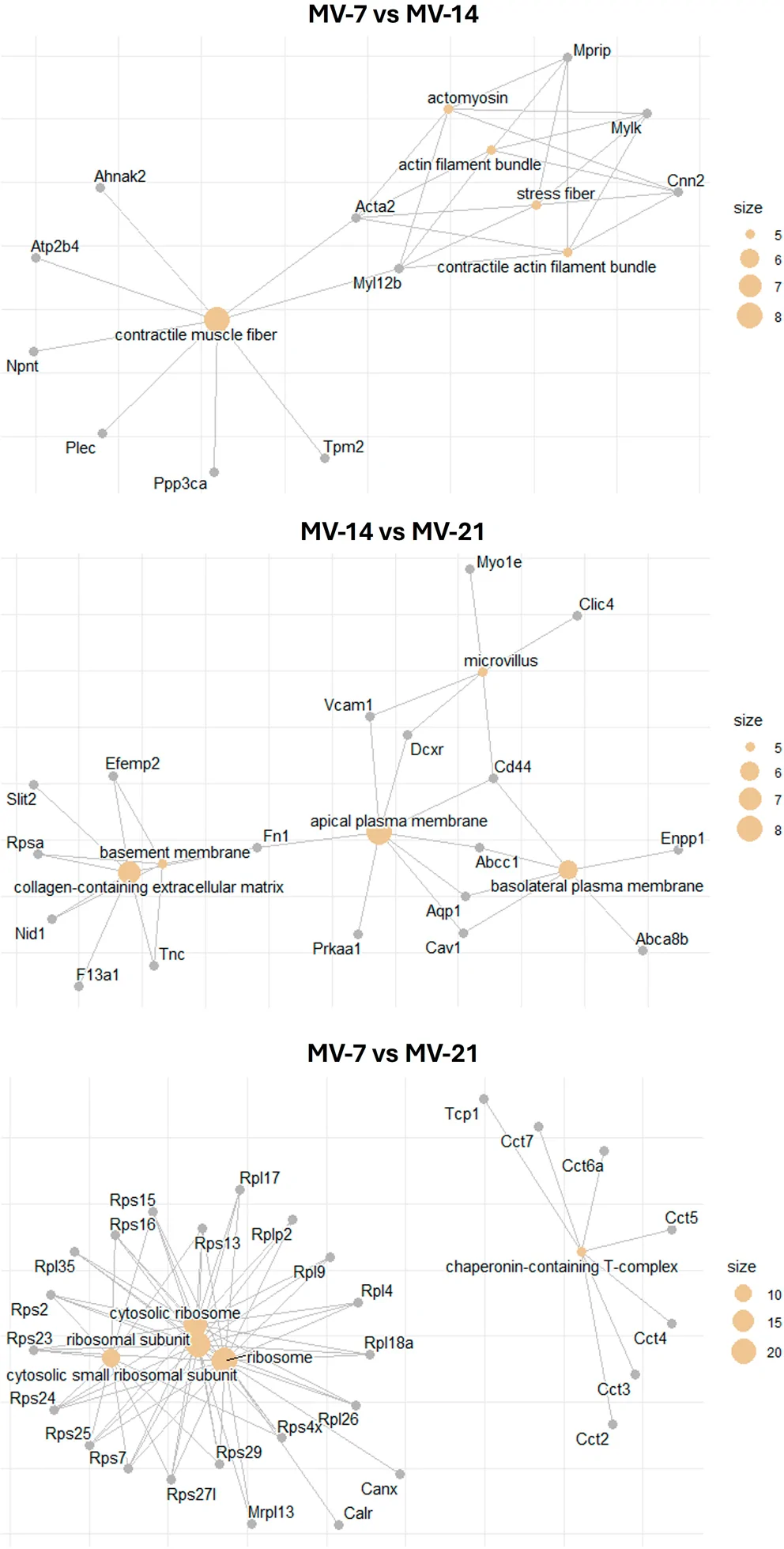
Gene Ontology (GO) network analysis of unique proteins identified from Venn diagram comparisons. Matrix vesicles (MVs) were grouped according to the day of isolation: day 7 (MV-7), day 14 (MV-14), and day 21 (MV-21). Pairwise comparisons between groups were performed to identify unique protein sets used for GO enrichment and network visualization. Node size reflects the number of proteins associated with each GO category.

## Discussion

Temporal biophysical, biochemical, and proteomic differences observed in MVs during days 7, 14, and 21 suggest dynamic functional remodeling associated with osteoblast maturation and the evolving demands of biomineralization. MVs produced in later stages become larger, mechanically more complex, and more competent for mineral formation. This progressive shift supports the concept that MVs are not static entities but rather dynamic structures whose composition and activity evolve as mineralization advances.

Indeed, similar modifications in vesicle morphology and composition have been associated with osteoblast maturation and increased mineralizing potential.^12,24^ Changes in vesicle size and surface charge are commonly associated with membrane remodeling, lipid reorganization, and protein loading. As osteoblasts progress toward a mineralizing phenotype, membranes become enriched in acidic phospholipids and sphingolipid-cholesterol domains that favor calcium binding and apatite nucleation.^10,12,25,26^ AFM phase images of MVs further suggest that maturation is accompanied by internal compartmentalization. The coexistence of stiffer and softer domains, particularly evident in MV-14 and MV-21, is compatible with progressive mineral accumulation and protein–lipid reorganization previously described during nucleation events inside vesicles.^12,25,27,28^

The progressive rise in TNAP activity from MV-7 to MV-21 strongly correlates with the enhanced ability of late vesicles to trigger mineral formation in vitro. TNAP is a key regulator of phosphate homeostasis because it hydrolyzes pyrophosphate (PPi), a potent inhibitor of hydroxyapatite formation, while increasing local inorganic phosphate (Pi) availability.^29^ Therefore, higher TNAP activity reduces inhibitory pressure and promotes nucleation, explaining the faster onset of turbidity observed for MV-21. The idea that enzymatic activity—rather than simple protein presence—defines vesicle competence has been repeatedly emphasized in mineralization models.^30–33^ In addition, TNAP was previously shown to be heterogeneously distributed among osteoblast-derived MVs isolated at day 14, with TNAP-positive vesicles representing only a minority subpopulation.^34^ These findings further support the existence of functionally distinct MVs subsets rather than homogeneous vesicle population, not only stage-dependent but also within the same differentiation period.

Despite the changes aforementioned, the abundance of most proteins associated with biomineralization remained relatively stable. Notable exceptions included ENPPs (ENPP1 and ENPP3) and ANXA6. ENPPs play pivotal role in regulating the extracellular PPi/Pi balance through ATP hydrolysis, while ANXA6 contributes to early MVs membrane dynamics for Ca^2+^ influx and crystal nucleation.^35–37^ The transient elevation of ENPP1 in MV-14 likely suggests a protection against premature mineralization regulating PPi levels before TNAP-mediated hydrolysis promotes mineral propagation.^38,39^ Complementing this, ENPP3 showed low levels during the ECM organization phase (days 7–14), followed by an elevation during active biomineralization (days 14–21), suggesting a shift from PPi-dominant inhibition to Pi-promoting crystallization.^38–41^ Similarly, ANXA6 was elevated on day 7 and lowered on day 21. This biphasic profile indicates changes in Ca^2+^ requirements by MVs and a prevention of Ca^2+^ overload at mineral propagation phase. Like ANXA5, which primes early mineralization before declining, this supports annexin coordination in phased events.^36,37,42,43^ Together, this modulation of ENPP1, ENPP3, and ANXA6 provides a temporal perspective for previously reported mineralization-associated mechanisms and reinforces the concept that the core protein machinery governing biomineralization is dynamically and finely regulated throughout osteoblast differentiation.

EVs biogenesis routes remain heterogeneous. The identification of proteins associated with both ectosome shedding and endosomal pathways across all time points indicates that osteoblasts likely release a heterogeneous population of EVs throughout differentiation, as widely recognized in the extracellular vesicle field.^44^ Further studies are required to elucidate the biogenesis pathways, turnover, and half-time dynamics of these distinct MVs populations.

The enrichment patterns observed in the differential proteomic comparisons reinforce the maturation model. Early transitions were associated with cytoskeletal organization and adhesion-related components, consistent with mechanisms required for vesicle budding, trafficking, and anchorage to the extracellular matrix.^45,46^ In later stages, MVs appear to function as anchoring and mineral-nucleating platforms, consistent with enrichment in proteins linked to metabolic activity and tissue remodeling, which are commonly associated with active mineralization and matrix organization.^30^ At 21 days, we also observed an enrichment of ribosomal proteins, chaperones, and translation-related categories. Although EVs are not capable of autonomous protein synthesis, these molecules are frequently detected in vesicle cargo and are thought to contribute to structural stability, stress protection, or regulatory roles.^47–49^ However, because ribosomal proteins in EVs can also result from coisolation, passive packaging, or cell-state-dependent cargo selection,^13,50,51^ their functional interpretation should remain cautious until supported by direct mechanistic evidence.

Furthermore, the increased abundance of polypeptides associated with integrins and proteoglycans suggests that latter stage vesicles acquire a greater specialization for matrix interaction, which is likely important for efficient propagation of minerals beyond the vesicular lumen.^16,46,52,53^ This interpretation is supported by our collagen-binding assay, in which all MV groups retained the ability to interact with type I collagen, the main organic component of the bone matrix.^54^ These findings indicate that, during biomineralization, MVs progressively enhance proteins associated with the interaction with bone ECM.

In conclusion, our findings demonstrate that distinct MVs are produced throughout osteoblast differentiation and exhibit stage-dependent biophysical, biochemical, and proteomic characteristics. During the early stage (days 7–14), MVs displayed proteomic features associated with ECM organization and the establishment of a mineralization-prone microenvironment. In contrast, during the later stage (days 14–21), pro-mineralization features become more prominent, with MVs exhibiting proteomic changes associated with calcium response and ECM interactions, together with increased TNAP activity, collagen anchoring, and mineralization capacity. Collectively, these findings support the concept that MVs properties and cargo are dynamically remodeled during osteoblast differentiation and the progression of biomineralization. Importantly, the present data do not establish a direct causal relationship between individual proteomic changes and MVs mineralization; the convergence of proteomic, biochemical, biophysical, and functional findings supports a model of stage-dependent functional remodeling of MVs during osteoblast differentiation. Mechanistic studies, such as selective inhibition, or modulation of individual candidate proteins, followed by assessment of MVs mineralization, will be necessary to establish causal relationships between specific molecular changes and MVs function.

## Supplementary Material



## Data Availability

The mass spectrometry proteomics data have been deposited to the ProteomeXchange Consortium via the PRIDE partner repository (http://www.ebi.ac.uk/pride/archive/) with the data set identifier PXD081367. Figure 1A and TOC were created with https://www.biorender.com/.

## Notes

Differentially expressed proteins identified by Limma-treat (FDR < 0.05, |fold change| > 2×) across pairwise temporal contrasts (MV-7, MV-14, and MV-21), tables organized according to the Venn diagram partition, tables organized by the direction of protein abundancy (UP/DOWN) and Venn diagram partition, and Gene ontology (GO) enrichment analysis on the sets of unique proteins identified across pairwise temporal contrasts (MV-7, MV-14, and MV-21) (PDF)

## Abbreviations

AFM: atomic force microscopy
ANXA: annexin
ATR-FTIR: attenuated total reflectance–Fourier transform infrared spectroscopy
ATP: adenosine triphosphate
ATP2B1: plasma membrane calcium transport ATPase1
ATP2B4: plasma membrane calcium transport ATPase
BP: biological process
CC: cellular compartment
CD: tetraspanins
CSGALNACT1: chondroitin sulfate *N*-acetylgalactosaminyltransferase 1
DLS: dynamic light scattering
ECM: extracellular matrix
ENPP: ectonucleotide pyrophosphatases/phosphodiesterases
ERK1: extracellular signal-regulated kinase 1
ERK2: extracellular signal-regulated kinase 2
EVs: extracellular vesicles
FC: fold change
FDR: false discovery rate
GO: gene ontology
H2BC14: H2B clustered histone 14
ITGB1: integrin beta-1
LAMP1: lysosome-associated membrane glycoprotein 1
LC-MS/MS: liquid chromatography-tandem mass spectrometry
MF: molecular function
MISEV: minimal information for studies of extracellular vesicles
MMP2: 72 kDa type IV collagenase also known as matrix metalloproteinase-2
MVs: matrix vesicles
NTA: nanoparticle tracking analysis
PCA: principal component analysis
PC1: principal component 1
PC2: principal component 2
PHOSPHO1: orphan phosphatase 1
Pi: inorganic phosphate
PPi: inorganic pyrophosphate
PTN: pleiotrophin
PVR: poliovirus receptor
SLC20A2: sodium-dependent phosphate transporter 2
SD: standard deviation
SE: standard error
SEM: scanning electron microscopy
SMPD2: neutral sphingomyelinase
SET: SET nuclear proto-oncogene
TSG101: tumor susceptibility gene 101 protein
TMT: tandem mass tags
TNAP: tissue-nonspecific alkaline phosphatase
VPS36: vacuolar protein-sorting-associated protein 36
VTN: vitronectin
