# Supplementary material for: Stage-Specific Proteomic Signatures of Matrix Vesicles Support Functional Transitions during Biomineralization
Source: J Proteome Res. 2026 Oct 20;25(11):6380–91. doi: 10.1021/acs.jproteome.6c00508 (PMC13750072; doi:10.1021/acs.jproteome.6c00508)
Supplement: Supplementary file 1 [file pr6c00508_si_001.pdf]

Supporting Information 1

Stage-Specific Proteomic Signatures of Matrix Vesicles Support Functional Transitions during Biomineralization

Juçara Gastaldi Cominal[1]; Larwsk Hayann[1]; Maryanne Trafani de Melo[1]; Iara Souza Lima[1]; Angie Lissette Atoche Puelles[1]; Lucas Fabricio Bahia Nogueira[1]; Saida Mebarek[3]; Ana Paula Ramos[1]; José Luis Millán*[4]; Massimo Bottini*[2,4]; and Pietro Ciancaglini*[1]

[1]Departamento de Química, Faculdade de Filosofia, Ciências e Letras de Ribeirão Preto, Universidade de São Paulo, 14040-901, Ribeirão Preto, Brasil.

[2]Department of Experimental Medicine, University of Rome Tor Vergata, 00133, Rome, Italy.

[3]Université de Lyon1, UMR CNRS 5246, 69 622 Villeurbanne, France.

[4]Sanford Children's Health Research Center, Sanford Burnham Prebys Medical Discovery Institute, 92037 La Jolla CA, USA.

* Corresponding authors: pietro@ffclrp.usp.br (Pietro Ciancaglini); massimo.bottini@uniroma2.it (Massimo Bottini) or millan@sbpdiscovery.org (José Luis Millán)

Table of Contents

- **Supplemental 1**

(A) Differentially expressed proteins identified by Limma-treat (FDR < 0.05, |fold change| > 2×) across pairwise temporal contrasts (MV-7, MV-14, MV-21). Tables are organized according com Venn diagram partition (showed in Figure 4C).

(B) Differentially expressed proteins identified by Limma-treat (FDR < 0.05, |fold change| > 2×) across pairwise temporal contrasts (MV-7, MV-14, MV-21). Tables are organized by direction of protein abundancy (UP/DOWN) and Venn diagram partition (showed in Figure 4D) (PDF).

- **Supplemental 2**

Gene ontology (GO) enrichment analysis on the sets of unique proteins identified across pairwise temporal contrasts (MV-7, MV-14, MV-21) (PDF).

A. Differentially expressed proteins identified by Limma-treat (FDR < 0.05, |fold change| > 2×) across pairwise temporal contrasts (MV-7, MV-14, MV-21). Tables are organized according com Venn diagram partition (showed in Figure 4C).

1. Common proteins (all 3 contrasts)

n = 5 proteins | Criteria: FDR < 0.05, |log2FC| > 1 (fold change > 2×).

Proteins
Csgalnact1
H2bc14
Ptn
Pvr
Set

1.1. MV-7 *vs* MV-14

n = 254 proteins | Criteria: FDR < 0.05, |log2FC| > 1 (fold change > 2×).

Exclusive (n=73)	Shared with MV-14 *vs* MV-21 (n=89)	Shared MV-7 *vs* MV-21 (n=87)
Acat1	Adamts16	Anpep
Acta2	Adamts5	Aspn
Actr1a	Ak1	Atp5po
Ahnak2	Anxa6	Bgn
Aldh7a1	Anxa8	Btf3
Atp2b4	Ap2s1	Btf3l4
Bop1	Ass1	Cltb
C4b	Atp1b1	Cltc
Cald1	Basp1	Col11a1
Cdh11	Bdh2	Csde1
Cemip2	Cemip	Edc4
Clec2d	Cgas	Eif4g1
Cnn2	Cnp	Fech
Cpne2	Col12a1	Fmn1
Cryz	Col6a5	Galnt1
Csnk2a2	Col6a6	Galnt7
Cspg4	Cpne8	Gas6
Dnpep	Cthrc1	Gmppa
Dpep1	Cyp2f2	Gng12
Edil3	Dhcr24	Gpc1
Ehd2	Efemp1	Gpc4
Eif3j1	Eif2b5	Gpnmb
Eif6	Enpp3	H1-4
Entpd3	Fat1	Hdlbp
Epha2	Fbn2	Itga10
Fam3c	Ftl1	Lman1
Fcgrt	Gm49394	Lyar
Fst	Gsdmd	Map3k4
Gar1	H1-0	Mix23
Gnptab	H2ac15	Mrpl28
Gpi	H2ax	Naa15
Grhl2	H2az1	Ntn1
Gyg1	H2bc1	Ogn

Exclusive (n=73)	Shared with MV-14 *vs* MV-21 (n=89)	Shared MV-7 *vs* MV-21 (n=87)
Hfe	H3c11	Oxct1
Hnrnpa2b1	H4c16	Pa2g4
Lman2	Hist2h2aa2	Polr3a
Lox	Hspd1	Ppp2r5d
Man2a1	Htra1	Prelp
Mesd	Ifi202	Psmd13
Mprip	Igf2	Psmd4
Mrc2	Igsf10	Psmd6
Mrpl11	Igsf8	Rpl10
Mrpl58	Il1rap	Rpl12
Mrps30	Kdsr	Rpl13
Myl12b	Ldhb	Rpl19
Myl6	Lix1l	Rpl21
Mylk	Loxl1	Rpl22l1
Naglu	Ly75	Rpl23
Nans	Macroh2a2	Rpl23a
Npc2	Marcksl1	Rpl24
Npnt	Mfge8	Rpl27
Nucb1	Mrps26	Rpl27a
Pcolce	Naalad2	Rpl29
Pepd	Ncl	Rpl3
Plec	Niban1	Rpl30
Plpp3	Npm1	Rpl31
Ppp3ca	Nrdc	Rpl32
Psap	Nsdhl	Rpl34
Psmc1	P10404	Rpl36
Psmc5	P2ry10b	Rpl36a
Ptpn13	Pde8a	Rpl37
Rab9a	Pip4p2	Rpl37a
Rps11	Pltp	Rpl38
Rrm1	Polr2a	Rpl8
Sdf4	Polr2b	Rplp0
Sec13	Polr2c	Rps8
Slc16a1	Polr2i	Rps9
Smpd2	Psmb5	Sap18
Snx5	Psmb8	Scara5
Srp72	Ptma	Slc2a1
Suclg1	Rab21	Snd1
Tmed5	Rbp1	Snrpa
Tpm2	S100a10	Srpx
	S100a13	Srpx2
	S1pr1	Srsf3
	Scrn1	Ssr3
	Serpine2	Tenm4
	Sgcb	Thy1
	Slit3	Tmed10
	Sord	Tmed4
	Steap3	Tmed7
	Thbs1	Tmed9
	Thsd4	Top2b
	Tnfrsf10b	Tsr1
	Tnxb	Wdr6
	Tpm1	Wfs1

Exclusive (n=73)	Shared with MV-14 *vs* MV-21 (n=89)	Shared MV-7 *vs* MV-21 (n=87)
	Utp18	Xpnpep2
	Vcan	
	Vim	

1.2. MV-14 *vs* MV-21

n = 200 proteins | Criteria: FDR < 0.05, |log2FC| > 1 (fold change > 2×).

Exclusive (n=61)	Common in MV-7 vs MV-21 (n=45)
Abca8b	Acp3
Abcc1	Angptl2
Acadl	Ap2m1
Aqp1	Bst2
Asah1	Cd200
C3	Col1a1
Calhm5	Col5a1
Calm1	Cul7
Cav1	Enpep
Ccn2	Fas
Cd44	Fbln1
Clic4	Hp
Dcxr	Ifit1
Dstn	Mcm3
Ece1	Ncbp1
Efemp2	Nfix
Ehd1	Nmd3
Enpp1	Nt5e
Ephx1	Obsl1
F13a1	Pcca
Fabp5	Pdpn
Fn1	Prss23
Gpx1	Raet1e
Hnrnpf	Rangap1
IGK	Rpl10a
Igfbp4	Rpl11
Inf2	Rpl13a
Ipo11	Rpl14
Mdh2	Rpl15
Mme	Rpl18
Mtpn	Rpl28
Myo1e	Rpl35a
Nid1	Rpl5
Npepl1	Rpl6
Nt5c2	Rpl7
P2rx7	Rpl7a

Exclusive (n=61)	Common in MV-7 vs MV-21 (n=45)
Pappa	Slc39a14
Pdcd6	Smarca5
Pdia4	Sod2
Pef1	Sphk1
Pfkl	Taldo1
Polr2h	Tgfbi
Ppt1	Tgm2
Prkaa1	Top1
Ptpra	Trim28
Ptx3	
Rap1b	
Rpsa	
Serpinb6	
Sgcd	
Slit2	
Slk	
Slpi	
Smpdl3b	
Tagln2	
Tmem43	
Tnc	
Trmt1l	
Ube2o	
Vcam1	
Ywhaq	

1.3. MV-7 *vs* MV-21

n = 200 proteins | Criteria: FDR < 0.05, |log2FC| > 1 (fold change > 2×).

Exclusive (n=87)
Aacs
Adam10
Arih1
Arsb
Atl3
Atp5f1a
Atp5f1b
Atp5f1e
Atp6v1d

Exclusive (n=87)
Bloc1s5
Bri3bp
Calr
Canx
Caprin1
Cct2
Cct3
Cct4
Cct5
Cct6a
Cct7
Cd109
Cd47
Clta
Cnn3
Cp
Cxcl12
Cyb5b
Ddah2
Dock7
Eftud2
Eif3e
Eif3l
Fhip2a
Fhod1
Fut8
Gns
Gtpbp1
Hgf
Ifi204
Ifitm3
Kars1
Lgals3
Lta4h
Maged2
Manf
Mccc1
Mfap5
Mob1b
Mrpl13
Mvk
Myh14
Myh9
Nedd4
Osbp

Exclusive (n=87)
Plbd2
Pof1b
Psmc2
Psmd11
Psmd7
Rapgef1
Rpl17
Rpl18a
Rpl26
Rpl35
Rpl4
Rpl9
Rplp2
Rps13
Rps15
Rps16
Rps2
Rps23
Rps24
Rps25
Rps27l
Rps29
Rps3a
Rps4x
Rps7
Rtn3
Rtn4
Serinc1
Smc3
Snrnp70
Tcp1
Tmed3
Tmem87b
Tom1l2
Tpst1
Usp9x
VTN

B. Differentially expressed proteins identified by Limma-treat (FDR < 0.05, |fold change| > 2×) across pairwise temporal contrasts (MV-7, MV-14, MV-21). Tables are organized by direction of protein abundancy (UP/DOWN) and Venn diagram partition (showed in Figure 4D).

Abbreviations: log2FC = log2 fold change; FDR = false discovery rate (Benjamini-Hochberg).

2. UP Proteins

2.1. Common proteins (all 3 contrasts)

n = 1 protein | Criteria: FDR < 0.05, |log2FC| > 1 (fold change > 2×).

Protein	MV-7 *vs* MV-14		MV-14 *vs* MV-21		MV-7 *vs* MV-21	
	log2FC	FDR	log2FC	FDR	log2FC	FDR
Ptn	1.675	1.15e-04	1.304	3.13e-02	2.979	3.63e-08

2.2. MV-7 *vs* MV-14

Exclusive proteins

n = 77 proteins | Criteria: FDR < 0.05, |log2FC| > 1 (fold change > 2×). Colored cells indicate statistically significant values, and gray cells represent not significant findings.

Protein	MV-7 *vs* MV-14		MV-14 *vs* MV-21		MV-7 *vs* MV-21	
	log2FC	FDR	log2FC	FDR	log2FC	FDR
Acat1	1.695	1.37e-03	-0.526	1.00e+00	1.168	6.24e-01
Acta2	1.358	1.51e-02	-0.838	1.00e+00	0.52	1.00e+00
Actr1a	1.545	6.17e-03	-0.337	1.00e+00	1.208	4.02e-01
Ak1	2.224	1.13e-05	-1.601	3.22e-03	0.623	1.00e+00
Aldh7a1	1.355	4.98e-03	-1.087	9.08e-01	0.268	1.00e+00
Anxa6	1.42	1.01e-03	-1.542	1.69e-04	-0.121	1.00e+00
Anxa8	2.565	2.97e-06	-2.083	7.36e-05	0.482	1.00e+00
Ass1	1.276	3.66e-02	-1.328	1.80e-02	-0.052	1.00e+00
Atp2b4	1.316	1.78e-02	-1.017	1.00e+00	0.299	1.00e+00
Cald1	1.476	1.25e-03	-0.829	1.00e+00	0.647	1.00e+00
Cdh11	1.287	2.48e-02	-0.744	1.00e+00	0.543	1.00e+00
Cemip2	1.786	3.50e-05	-0.94	1.00e+00	0.845	1.00e+00
Cnn2	1.268	2.23e-02	-1.074	1.00e+00	0.195	1.00e+00
Cnp	1.831	4.46e-06	-1.414	1.42e-03	0.417	1.00e+00
Col12a1	1.56	1.58e-03	-1.556	1.95e-03	0.004	1.00e+00
Cspg4	1.347	1.61e-02	-0.211	1.00e+00	1.136	5.31e-01
Ehd2	1.431	3.50e-03	-0.816	1.00e+00	0.614	1.00e+00
Eif3j1	1.226	3.13e-02	-0.822	1.00e+00	0.404	1.00e+00
Eif6	1.555	1.87e-03	-1.183	4.20e-01	0.372	1.00e+00
Entpd3	1.562	1.05e-03	-0.693	1.00e+00	0.869	1.00e+00
Epha2	1.751	5.92e-05	-1.256	8.14e-02	0.495	1.00e+00
Fat1	2.098	2.42e-06	-1.354	1.45e-02	0.745	1.00e+00
Fbn2	2.722	2.95e-07	-2.86	1.70e-07	-0.138	1.00e+00
Fcgrt	1.745	1.52e-04	-0.531	1.00e+00	1.214	2.13e-01
Fst	1.459	5.45e-03	-0.248	1.00e+00	1.211	2.32e-01
Grhl2	2.104	1.67e-06	-0.956	1.00e+00	1.148	3.78e-01
Gsdmd	1.692	7.19e-05	-2.118	1.77e-06	-0.426	1.00e+00
Gyg1	1.944	2.30e-04	-1.166	8.07e-01	0.778	1.00e+00
H1-0	2.986	1.97e-08	-2.289	2.07e-07	0.697	1.00e+00
Hfe	2.422	5.48e-07	-1.257	9.37e-02	1.165	3.66e-01
Hnrnpa2b1	1.285	3.52e-02	-0.782	1.00e+00	0.503	1.00e+00
Htra1	2.319	3.97e-07	-1.262	4.89e-02	1.057	1.00e+00
Ly75	1.735	1.02e-04	-2.192	2.38e-06	-0.457	1.00e+00
Mprip	1.307	3.58e-02	-0.341	1.00e+00	0.965	1.00e+00
Mrc2	1.267	2.79e-02	-1.046	1.00e+00	0.221	1.00e+00

Protein	MV-7 *vs* MV-14		MV-14 *vs* MV-21		MV-7 *vs* MV-21	
	log2FC	FDR	log2FC	FDR	log2FC	FDR
Mrpl11	1.367	1.96e-02	-0.725	1.00e+00	0.642	1.00e+00
Mrpl58	1.561	2.90e-04	-0.509	1.00e+00	1.052	1.00e+00
Mrps26	1.813	3.15e-03	-1.67	1.30e-02	0.143	1.00e+00
Mrps30	2.328	6.51e-05	-1.076	1.00e+00	1.252	4.78e-01
Myl12b	1.298	2.54e-02	-0.133	1.00e+00	1.165	2.93e-01
Myl6	2.146	3.08e-06	-1.295	5.73e-02	0.852	1.00e+00
Mylk	1.644	1.15e-04	-1.238	8.14e-02	0.406	1.00e+00
Nans	1.356	2.95e-03	-0.931	1.00e+00	0.425	1.00e+00
Niban1	1.809	1.43e-05	-1.862	1.09e-05	-0.053	1.00e+00
Npnt	1.699	7.01e-05	-0.462	1.00e+00	1.237	8.09e-02
P10404	2.163	4.48e-05	-2.553	5.41e-06	-0.39	1.00e+00
P2ry10b	1.468	4.44e-03	-1.872	5.10e-05	-0.404	1.00e+00
Pcolce	1.716	3.50e-04	-1	1.00e+00	0.716	1.00e+00
Pde8a	2.466	2.88e-07	-2.187	1.12e-06	0.279	1.00e+00
Pip4p2	1.585	2.46e-04	-1.73	5.10e-05	-0.145	1.00e+00
Plec	1.447	1.39e-03	-0.681	1.00e+00	0.766	1.00e+00
Pltp	2.024	3.28e-05	-1.794	3.05e-04	0.23	1.00e+00
Ppp3ca	1.529	2.73e-04	-1.042	1.00e+00	0.488	1.00e+00
Psmb8	2.414	4.00e-04	-3.077	2.47e-05	-0.663	1.00e+00
Psmc1	1.327	5.26e-03	-0.183	1.00e+00	1.145	2.55e-01
Psmc5	1.256	3.81e-02	-0.688	1.00e+00	0.567	1.00e+00
Ptpn13	1.719	6.48e-03	-0.766	1.00e+00	0.953	1.00e+00
Rab21	1.383	3.58e-02	-1.657	1.62e-03	-0.274	1.00e+00
Rps11	1.456	9.16e-03	-0.176	1.00e+00	1.279	1.14e-01
Rrm1	1.328	2.56e-02	-0.964	1.00e+00	0.363	1.00e+00
S100a10	1.877	2.55e-05	-1.788	7.43e-05	0.089	1.00e+00
S100a13	2.226	3.08e-06	-2.669	3.35e-07	-0.443	1.00e+00
S1pr1	1.393	1.18e-02	-1.675	3.36e-04	-0.281	1.00e+00
Scrn1	2.219	1.08e-06	-1.72	8.59e-05	0.499	1.00e+00
Sec13	1.644	4.79e-05	-0.618	1.00e+00	1.025	1.00e+00
Serpine2	2.063	1.24e-06	-1.415	2.31e-03	0.647	1.00e+00
Sgcb	2.407	9.31e-04	-3.241	2.93e-05	-0.835	1.00e+00
Snx5	1.344	3.75e-03	-0.954	1.00e+00	0.389	1.00e+00
Sord	1.46	9.69e-04	-1.684	5.16e-05	-0.224	1.00e+00
Srp72	1.333	5.77e-03	-0.413	1.00e+00	0.92	1.00e+00
Suclg1	1.299	3.45e-02	-0.822	1.00e+00	0.477	1.00e+00
Thbs1	2.375	1.13e-06	-1.762	1.55e-04	0.614	1.00e+00
Thsd4	3.074	6.26e-07	-1.936	4.07e-04	1.139	9.59e-01
Tnfrsf10b	1.489	3.71e-02	-1.809	2.21e-03	-0.321	1.00e+00
Tpm1	1.95	3.23e-05	-1.376	2.76e-02	0.574	1.00e+00
Tpm2	1.719	5.18e-04	-1.049	1.00e+00	0.67	1.00e+00
Vim	1.502	2.72e-03	-1.864	5.10e-05	-0.362	1.00e+00

Shared proteins compared with MV-7 *vs* MV-21

n = 71 proteins |Criteria: FDR < 0.05, |log2FC| > 1 (fold change > 2×). Colored cells indicate statistically significant values, and gray cells represent not significant findings.

Protein	MV-7 *vs* MV-14		MV-14 *vs* MV-21		MV-7 *vs* MV-21	
	log2FC	FDR	log2FC	FDR	log2FC	FDR
Aspn	1.587	1.93e-03	0.498	1.00e+00	2.085	1.90e-05
Atp5po	1.516	4.38e-03	0.149	1.00e+00	1.665	8.29e-04
Bgn	2.807	1.51e-07	0.149	1.00e+00	2.956	4.98e-08
Btf3	2.627	1.87e-04	-0.088	1.00e+00	2.539	3.33e-04
Btf3l4	3.833	8.10e-06	-0.134	1.00e+00	3.7	1.44e-05
Cltb	1.73	7.13e-05	0.193	1.00e+00	1.924	1.27e-05
Cltc	2.122	2.49e-06	0.021	1.00e+00	2.143	2.22e-06
Col11a1	2.687	1.51e-07	0.177	1.00e+00	2.864	5.93e-08
Csde1	1.286	1.18e-02	-0.042	1.00e+00	1.244	3.10e-02
Edc4	1.296	3.51e-03	1.121	3.01e-01	2.417	3.37e-08
Eif4g1	1.833	1.87e-04	-0.279	1.00e+00	1.554	4.07e-03
Fech	1.858	1.81e-05	-0.248	1.00e+00	1.61	3.30e-04
Fmn1	2.606	1.58e-07	-0.258	1.00e+00	2.348	3.36e-07
Gas6	3.038	1.32e-05	-0.559	1.00e+00	2.479	2.02e-04
Gmppa	1.981	3.44e-04	-0.144	1.00e+00	1.838	1.30e-03
Gpc1	1.484	9.32e-03	0.535	1.00e+00	2.019	4.79e-05
Gpc4	2.125	7.74e-07	0.741	1.00e+00	2.866	3.37e-08
H1-4	1.417	2.92e-03	-0.012	1.00e+00	1.406	3.86e-03
Hdlbp	1.587	6.23e-04	-0.271	1.00e+00	1.316	3.43e-02
Itga10	2.435	4.49e-07	-1.051	1.00e+00	1.384	9.59e-03
Lyar	2.819	1.51e-07	-0.247	1.00e+00	2.572	2.35e-07
Map3k4	1.442	4.32e-03	0.317	1.00e+00	1.759	9.09e-05
Mrpl28	2.387	2.55e-05	-0.536	1.00e+00	1.851	1.36e-03
Naa15	1.495	4.18e-04	-0.165	1.00e+00	1.33	8.01e-03
Ntn1	1.809	8.10e-06	0.275	1.00e+00	2.084	6.90e-07
Ogn	2.513	1.51e-07	-0.614	1.00e+00	1.899	5.11e-06
Oxct1	1.256	2.77e-02	0.081	1.00e+00	1.337	6.38e-03
Pa2g4	2.046	4.94e-04	-0.25	1.00e+00	1.796	3.88e-03
Ppp2r5d	1.329	1.61e-02	0.06	1.00e+00	1.389	6.54e-03
Prelp	1.659	1.69e-03	-0.186	1.00e+00	1.472	1.55e-02
Psmd13	1.267	4.35e-02	0.512	1.00e+00	1.779	3.61e-05
Psmd4	1.286	3.42e-02	0.02	1.00e+00	1.306	2.70e-02
Psmd6	1.306	1.74e-02	-0.02	1.00e+00	1.285	2.76e-02
Rpl10	2.325	6.56e-07	0.441	1.00e+00	2.767	6.20e-08
Rpl12	2.173	7.50e-06	1.344	5.05e-02	3.517	3.37e-08
Rpl13	1.978	3.02e-05	0.639	1.00e+00	2.617	4.38e-07
Rpl19	2.525	4.73e-06	0.591	1.00e+00	3.116	2.96e-07
Rpl21	1.378	3.51e-03	1.075	1.00e+00	2.453	8.22e-08
Rpl22l1	2.081	3.13e-06	-0.207	1.00e+00	1.874	2.22e-05
Rpl23	2.145	5.48e-07	0.069	1.00e+00	2.214	2.70e-07
Rpl23a	2.761	1.08e-06	-0.046	1.00e+00	2.715	1.21e-06
Rpl24	2.416	2.91e-07	0.134	1.00e+00	2.55	8.44e-08
Rpl27	1.229	3.39e-02	1.105	5.76e-01	2.335	7.49e-08
Rpl27a	1.629	1.48e-04	0.886	1.00e+00	2.514	1.03e-07
Rpl29	2.35	3.24e-03	0.558	1.00e+00	2.908	2.93e-04
Rpl3	1.342	5.85e-03	0.934	1.00e+00	2.276	1.92e-07

Protein	MV-7 *vs* MV-14		MV-14 *vs* MV-21		MV-7 *vs* MV-21	
	log2FC	FDR	log2FC	FDR	log2FC	FDR
Rpl30	1.425	4.79e-02	0.645	1.00e+00	2.07	1.36e-04
Rpl31	1.772	1.42e-04	0.523	1.00e+00	2.295	2.22e-06
Rpl32	1.329	4.99e-03	0.743	1.00e+00	2.072	4.38e-07
Rpl34	2.277	1.28e-05	0.74	1.00e+00	3.017	2.83e-07
Rpl36	1.432	1.38e-03	1.124	5.15e-01	2.557	6.09e-08
Rpl36a	2.208	5.35e-07	0.829	1.00e+00	3.038	3.12e-08
Rpl37	1.79	9.39e-05	1.166	4.78e-01	2.956	6.20e-08
Rpl37a	2.151	1.12e-05	0.814	1.00e+00	2.964	1.28e-07
Rpl38	2.706	4.94e-07	0.558	1.00e+00	3.263	5.93e-08
Rpl8	1.631	1.71e-05	0.719	1.00e+00	2.349	5.93e-08
Rplp0	2.048	2.97e-06	1.219	1.32e-01	3.267	3.12e-08
Rps8	1.58	1.67e-04	-0.259	1.00e+00	1.321	1.19e-02
Rps9	1.384	3.58e-02	0.15	1.00e+00	1.533	6.32e-03
Sap18	2.076	3.08e-06	0.514	1.00e+00	2.59	1.22e-07
Scara5	1.416	1.26e-03	0.035	1.00e+00	1.45	7.95e-04
Snd1	2.055	1.71e-06	-0.471	1.00e+00	1.584	2.39e-04
Snrpa	1.276	1.83e-02	0.059	1.00e+00	1.335	6.48e-03
Srpx	2.99	1.54e-07	-0.802	1.00e+00	2.189	6.49e-06
Srpx2	1.47	2.46e-04	0.099	1.00e+00	1.569	6.27e-05
Srsf3	1.369	4.99e-03	0.478	1.00e+00	1.847	1.05e-05
Ssr3	1.517	1.55e-03	-0.166	1.00e+00	1.35	2.00e-02
Tenm4	1.878	1.42e-05	-0.588	1.00e+00	1.29	3.68e-02
Top2b	1.54	2.46e-04	0.98	1.00e+00	2.519	6.20e-08
Tsr1	1.278	2.90e-02	0.554	1.00e+00	1.832	1.44e-05
Wdr6	2.512	2.72e-06	-0.291	1.00e+00	2.221	1.80e-05

2.3. MV-14 *vs* MV-21

Exclusive UP proteins

n = 82 proteins | Criteria: FDR < 0.05, |log2FC| > 1 (fold change > 2×). Colored cells indicate statistically significant values, and gray cells represent not significant findings.

Protein	MV-7 *vs* MV-14		MV-14 *vs* MV-21		MV-7 *vs* MV-21	
	log2FC	FDR	log2FC	FDR	log2FC	FDR
Acadl	-0.656	1.00e+00	1.706	3.24e-04	1.05	1.00e+00
Adamts16	-3.05	4.44e-08	2.719	9.60e-08	-0.331	1.00e+00
Adamts5	-3.576	1.51e-07	3.252	2.68e-07	-0.324	1.00e+00
Ap2s1	-1.974	1.42e-04	1.823	6.15e-04	-0.151	1.00e+00
Aqp1	-1.147	1.29e-01	1.275	7.10e-03	0.128	1.00e+00
Asah1	-1	1.00e+00	1.458	2.54e-04	0.459	1.00e+00
Atp1b1	-2.025	6.59e-06	1.88	2.93e-05	-0.145	1.00e+00
Basp1	-2.683	3.06e-06	1.77	1.82e-03	-0.914	1.00e+00
Bdh2	-1.508	1.01e-03	1.45	2.74e-03	-0.059	1.00e+00
Ccn2	-0.672	1.00e+00	1.674	3.72e-05	1.002	1.00e+00
Cemip	-1.29	1.38e-02	2.006	1.41e-06	0.716	1.00e+00
Cgas	-1.429	2.05e-02	1.886	1.72e-04	0.457	1.00e+00
Col6a5	-2.091	1.46e-06	2.35	3.32e-07	0.259	1.00e+00
Col6a6	-3.223	2.95e-07	2.788	1.41e-06	-0.435	1.00e+00
Cpne8	-2.884	1.51e-07	2.844	1.35e-07	-0.04	1.00e+00
Cthrc1	-3.538	1.60e-06	3.967	5.43e-07	0.429	1.00e+00

Protein	MV-7 *vs* MV-14		MV-14 *vs* MV-21		MV-7 *vs* MV-21	
	log2FC	FDR	log2FC	FDR	log2FC	FDR
Cyp2f2	-1.422	1.64e-03	1.258	4.05e-02	-0.164	1.00e+00
Dhcr24	-1.425	7.90e-03	1.53	2.17e-03	0.106	1.00e+00
Efemp1	-3.009	1.57e-05	3.092	1.49e-05	0.083	1.00e+00
Efemp2	-0.93	1.00e+00	1.587	3.35e-05	0.657	1.00e+00
Eif2b5	-1.435	3.72e-04	1.529	9.73e-05	0.094	1.00e+00
Enpp1	-1.169	2.38e-01	1.63	1.80e-04	0.461	1.00e+00
Enpp3	-2.799	1.24e-06	2.656	2.88e-06	-0.143	1.00e+00
Fn1	-0.782	1.00e+00	1.549	3.01e-03	0.767	1.00e+00
Ftl1	-3.144	1.51e-07	2.674	7.14e-07	-0.47	1.00e+00
Gm49394	-3.332	7.74e-07	3.257	1.12e-06	-0.075	1.00e+00
H2ac15	-2.405	4.41e-05	3.624	3.35e-07	1.219	6.24e-01
H2ax	-3.186	7.05e-04	4.408	2.72e-05	1.222	1.00e+00
H2az1	-2.879	7.21e-03	4.752	5.34e-05	1.873	2.55e-01
H2bc1	-3.412	6.10e-07	4.811	4.13e-08	1.399	1.31e-01
H3c11	-2.855	3.22e-04	4.547	2.17e-06	1.691	1.23e-01
H4c16	-3.128	2.38e-05	4.276	9.23e-07	1.148	1.00e+00
Hist2h2aa2	-3.105	1.93e-05	4.587	3.35e-07	1.481	1.99e-01
Hspd1	-1.748	2.52e-04	2.479	1.23e-06	0.731	1.00e+00
Ifi202	-3.031	1.51e-07	2.97	1.21e-07	-0.06	1.00e+00
Igf2	-4.077	1.82e-06	3.169	3.87e-05	-0.907	1.00e+00
Igsf10	-1.754	8.68e-06	2.471	8.59e-08	0.718	1.00e+00
Igsf8	-2.016	1.93e-05	1.848	1.00e-04	-0.168	1.00e+00
Il1rap	-1.844	1.00e-05	1.512	6.61e-04	-0.332	1.00e+00
Ipo11	-1.121	6.17e-01	1.541	1.58e-03	0.42	1.00e+00
Kdsr	-1.379	2.17e-02	1.338	4.73e-02	-0.04	1.00e+00
Ldhb	-3.314	4.62e-07	2.682	6.13e-06	-0.632	1.00e+00
Lix1l	-1.661	1.94e-04	2.091	4.13e-06	0.431	1.00e+00
Loxl1	-2.484	3.51e-07	2.891	8.59e-08	0.407	1.00e+00
Macroh2a2	-2.136	1.46e-06	2.421	3.13e-07	0.285	1.00e+00
Marcksl1	-2.938	7.08e-05	2.443	8.48e-04	-0.495	1.00e+00
Mfge8	-2.433	4.49e-07	1.476	2.49e-03	-0.957	1.00e+00
Myo1e	-0.54	1.00e+00	1.34	1.44e-02	0.8	1.00e+00
Naalad2	-2.055	6.45e-06	1.86	4.16e-05	-0.195	1.00e+00
Ncl	-1.985	1.68e-05	1.547	1.98e-03	-0.439	1.00e+00
Nid1	-1.017	1.00e+00	1.356	8.06e-03	0.339	1.00e+00
Npm1	-2.279	6.56e-07	1.348	1.45e-02	-0.931	1.00e+00
Nrdc	-2.416	2.91e-07	2.207	8.75e-07	-0.209	1.00e+00
Nsdhl	-1.767	2.83e-03	1.704	5.81e-03	-0.062	1.00e+00
Nt5c2	-0.982	1.00e+00	1.36	4.42e-02	0.378	1.00e+00
Pappa	-1.258	2.61e-01	2.034	1.38e-04	0.776	1.00e+00
Pdcd6	-0.786	1.00e+00	1.319	1.53e-02	0.533	1.00e+00
Pef1	-1.082	9.77e-01	2.082	8.75e-06	1	1.00e+00
Pfkl	-0.631	1.00e+00	1.747	5.48e-05	1.116	6.29e-01
Polr2a	-2.085	8.55e-06	2.035	1.62e-05	-0.051	1.00e+00
Polr2b	-1.995	4.50e-06	2.063	3.28e-06	0.068	1.00e+00
Polr2c	-2.107	2.53e-05	1.674	1.50e-03	-0.433	1.00e+00
Polr2h	-1.03	1.00e+00	1.442	9.70e-04	0.412	1.00e+00
Polr2i	-1.48	2.93e-04	1.822	4.85e-06	0.342	1.00e+00
Ppt1	-0.977	1.00e+00	1.49	3.13e-04	0.512	1.00e+00

Protein	MV-7 *vs* MV-14		MV-14 *vs* MV-21		MV-7 *vs* MV-21	
	log2FC	FDR	log2FC	FDR	log2FC	FDR
Psmb5	-1.948	2.97e-06	1.76	2.43e-05	-0.187	1.00e+00
Ptma	-1.729	1.67e-04	1.614	7.54e-04	-0.115	1.00e+00
Pvr	-3.248	1.51e-07	1.65	1.82e-03	-1.598	3.07e-03
Rbp1	-1.645	4.00e-04	2.072	8.75e-06	0.427	1.00e+00
Rpsa	-0.843	1.00e+00	1.936	1.32e-04	1.093	1.00e+00
Set	-3.168	3.06e-07	1.499	1.80e-02	-1.669	2.67e-03
Slit2	-0.559	1.00e+00	1.707	2.99e-05	1.148	2.82e-01
Slit3	-3.258	1.97e-08	3.032	4.13e-08	-0.227	1.00e+00
Slk	-0.411	1.00e+00	1.407	3.04e-03	0.996	1.00e+00
Steap3	-2.447	3.51e-07	2.262	9.53e-07	-0.186	1.00e+00
Tnc	-1.139	2.63e-01	1.241	4.05e-02	0.102	1.00e+00
Tnxb	-2.59	1.24e-06	2.185	1.62e-05	-0.405	1.00e+00
Trmt1l	-0.51	1.00e+00	1.636	1.69e-04	1.125	5.37e-01
Ube2o	-1.386	7.88e-02	1.946	4.21e-04	0.56	1.00e+00
Utp18	-2.003	5.62e-05	1.955	9.93e-05	-0.048	1.00e+00
Vcan	-1.768	2.99e-05	1.859	1.57e-05	0.091	1.00e+00
Ywhaq	-0.114	1.00e+00	1.319	1.32e-02	1.205	1.05e-01

Shared proteins compared with MV-7 *vs* MV-21

n = 31 proteins |Criteria: FDR < 0.05, |log2FC| > 1 (fold change > 2×). Colored cells indicate statistically significant values, and gray cells represent not significant findings.

Protein	MV-7 *vs* MV-14		MV-14 *vs* MV-21		MV-7 *vs* MV-21	
	log2FC	FDR	log2FC	FDR	log2FC	FDR
Angptl2	-0.754	1.00e+00	2.512	1.56e-07	1.758	3.01e-05
Ap2m1	-0.418	1.00e+00	2.297	8.75e-06	1.879	1.87e-04
Col1a1	1.047	1.00e+00	1.545	1.44e-02	2.592	4.33e-06
Col5a1	0.687	1.00e+00	1.417	1.64e-02	2.104	1.27e-05
Cul7	-0.432	1.00e+00	1.752	2.78e-05	1.32	1.25e-02
Fbln1	-0.006	1.00e+00	1.488	3.23e-04	1.482	3.45e-04
H2bc14	-3.604	6.42e-06	5.477	1.35e-07	1.873	2.08e-02
Mcm3	0.091	1.00e+00	1.81	5.34e-05	1.901	2.22e-05
Ncbp1	0.114	1.00e+00	1.611	3.08e-02	1.725	1.08e-02
Nfix	0.35	1.00e+00	1.585	2.13e-04	1.935	4.46e-06
Nmd3	0.101	1.00e+00	1.37	1.20e-02	1.471	2.27e-03
Obsl1	0.033	1.00e+00	1.322	3.50e-03	1.355	1.72e-03
Pcca	0.083	1.00e+00	1.502	4.97e-03	1.585	1.66e-03
Prss23	0.378	1.00e+00	1.924	9.53e-07	2.301	6.09e-08
Rangap1	-0.527	1.00e+00	1.873	3.64e-05	1.346	2.31e-02
Rpl10a	-0.336	1.00e+00	2.632	3.39e-07	2.296	1.97e-06
Rpl11	0.611	1.00e+00	1.695	1.62e-05	2.306	7.94e-08
Rpl13a	0.51	1.00e+00	1.511	9.45e-05	2.021	2.61e-07
Rpl14	0.459	1.00e+00	1.979	2.88e-06	2.438	9.76e-08
Rpl15	0.191	1.00e+00	1.918	4.16e-05	2.109	8.48e-06
Rpl18	0.023	1.00e+00	1.732	1.96e-04	1.755	1.52e-04
Rpl28	1.223	2.66e-01	1.611	2.55e-03	2.834	3.14e-07
Rpl35a	0.052	1.00e+00	2.228	2.33e-07	2.279	1.03e-07
Rpl5	0.152	1.00e+00	1.562	2.27e-03	1.714	3.81e-04
Rpl6	0.387	1.00e+00	1.312	3.65e-02	1.699	1.52e-04
Rpl7	0.694	1.00e+00	1.571	1.42e-04	2.265	1.69e-07

Protein	MV-7 *vs* MV-14		MV-14 *vs* MV-21		MV-7 *vs* MV-21	
	log2FC	FDR	log2FC	FDR	log2FC	FDR
Rpl7a	0.662	1.00e+00	2.182	2.38e-06	2.844	6.20e-08
Smarca5	0.059	1.00e+00	1.285	1.94e-02	1.345	5.71e-03
Tgfbi	-0.31	1.00e+00	1.842	1.50e-03	1.532	2.45e-02
Top1	0.176	1.00e+00	1.392	1.76e-03	1.568	9.97e-05
Trim28	0.373	1.00e+00	1.753	1.07e-05	2.126	2.83e-07

2.4. MV-7 *vs* MV-21

Exclusive proteins

n = 66 proteins | Criteria: FDR < 0.05, |log2FC| > 1 (fold change > 2×). Colored cells indicate statistically significant values, and gray cells represent not significant findings.

Protein	MV-7 vs MV-14		MV-14 vs MV-21		MV-7 vs MV-21	
	log2FC	FDR	log2FC	FDR	log2FC	FDR
Aacs	0.765	1.00e+00	0.83	1.00e+00	1.595	1.55e-04
Adam10	0.421	1.00e+00	1.012	1.00e+00	1.433	1.72e-03
Arih1	0.535	1.00e+00	0.707	1.00e+00	1.242	3.96e-02
Atp5f1a	1.114	6.96e-01	0.487	1.00e+00	1.601	8.29e-04
Atp5f1b	1.322	7.79e-02	0.401	1.00e+00	1.722	7.85e-04
Atp5f1e	1.184	1.96e-01	0.234	1.00e+00	1.418	4.14e-03
Bloc1s5	0.479	1.00e+00	1.077	9.61e-01	1.557	1.35e-04
Caprin1	1.277	4.70e-01	0.333	1.00e+00	1.609	3.96e-02
Cct2	1.166	3.04e-01	0.138	1.00e+00	1.303	3.61e-02
Cct3	0.898	1.00e+00	0.824	1.00e+00	1.722	1.44e-05
Cct4	0.812	1.00e+00	0.749	1.00e+00	1.561	1.77e-04
Cct5	0.884	1.00e+00	1.082	8.82e-01	1.966	1.00e-06
Cct6a	1.078	8.65e-01	0.372	1.00e+00	1.45	1.76e-03
Cct7	0.962	1.00e+00	0.654	1.00e+00	1.616	8.32e-04
Cd109	1.218	5.65e-02	0.193	1.00e+00	1.412	1.60e-03
Clta	1.017	1.00e+00	1.324	3.15e-01	2.341	8.93e-05
Cnn3	1.19	1.41e-01	0.261	1.00e+00	1.451	1.54e-03
Dock7	0.723	1.00e+00	0.612	1.00e+00	1.335	4.03e-02
Eftud2	0.178	1.00e+00	1.316	5.72e-02	1.495	4.12e-03
Eif3e	1.134	6.24e-01	0.473	1.00e+00	1.607	1.43e-03
Eif3l	0.583	1.00e+00	0.99	1.00e+00	1.573	4.91e-04
Fhip2a	1.159	3.07e-01	0.236	1.00e+00	1.395	6.77e-03
Fhod1	1.214	1.13e-01	0.492	1.00e+00	1.706	8.39e-05
Fut8	0.699	1.00e+00	0.782	1.00e+00	1.48	2.73e-02
Gtpbp1	0.704	1.00e+00	0.686	1.00e+00	1.39	4.14e-03
Hgf	0.917	1.00e+00	0.534	1.00e+00	1.451	1.22e-02
Kars1	1.146	2.91e-01	0.121	1.00e+00	1.266	3.35e-02
Maged2	1.012	1.00e+00	0.39	1.00e+00	1.402	1.21e-03
Mccc1	0.299	1.00e+00	1.107	9.08e-01	1.406	8.56e-03
Mob1b	0.207	1.00e+00	1.326	6.69e-02	1.533	3.86e-03
Mrpl13	0.482	1.00e+00	0.928	1.00e+00	1.409	7.08e-04
Mvk	0.216	1.00e+00	1.052	1.00e+00	1.267	1.23e-02
Myh14	0.929	1.00e+00	1.243	6.87e-02	2.171	6.90e-07
Myh9	0.2	1.00e+00	1.065	1.00e+00	1.265	3.73e-02
Nedd4	0.787	1.00e+00	0.899	1.00e+00	1.686	1.50e-05

Protein	MV-7 vs MV-14		MV-14 vs MV-21		MV-7 vs MV-21	
	log2FC	FDR	log2FC	FDR	log2FC	FDR
Osbp	1.114	7.03e-01	0.317	1.00e+00	1.432	8.48e-03
Pof1b	0.71	1.00e+00	1.201	8.98e-01	1.911	2.55e-03
Psmc2	1.194	1.10e-01	0.216	1.00e+00	1.41	2.22e-03
Psmd11	1.061	1.00e+00	0.388	1.00e+00	1.449	2.29e-03
Psmd7	0.853	1.00e+00	0.691	1.00e+00	1.544	2.39e-04
Rapgef1	1.129	3.48e-01	0.274	1.00e+00	1.403	2.16e-03
Rpl17	0.814	1.00e+00	1.286	2.10e-01	2.1	5.63e-05
Rpl18a	0.32	1.00e+00	1.264	1.02e-01	1.584	9.31e-04
Rpl26	1.14	5.12e-01	1.072	1.00e+00	2.213	3.10e-06
Rpl35	1.013	1.00e+00	0.436	1.00e+00	1.45	4.01e-04
Rpl4	1.263	5.57e-02	0.985	1.00e+00	2.248	8.37e-07
Rpl9	0.89	1.00e+00	0.879	1.00e+00	1.769	5.24e-05
Rplp2	1.14	2.50e-01	0.584	1.00e+00	1.724	1.50e-05
Rps13	0.913	1.00e+00	0.968	1.00e+00	1.881	1.36e-04
Rps15	1.225	2.60e-01	0.361	1.00e+00	1.586	3.13e-03
Rps16	0.198	1.00e+00	1.114	6.31e-01	1.312	1.48e-02
Rps2	0.645	1.00e+00	1.22	4.00e-01	1.865	2.66e-04
Rps23	1.225	6.82e-02	0.416	1.00e+00	1.641	9.77e-05
Rps24	0.572	1.00e+00	0.962	1.00e+00	1.534	4.20e-04
Rps25	0.591	1.00e+00	0.709	1.00e+00	1.3	1.23e-02
Rps27l	1.168	5.48e-01	0.757	1.00e+00	1.925	1.77e-04
Rps29	0.324	1.00e+00	1.131	4.41e-01	1.455	9.10e-04
Rps3a	1.013	1.00e+00	0.348	1.00e+00	1.361	2.55e-03
Rps4x	0.819	1.00e+00	0.444	1.00e+00	1.263	3.30e-02
Rps7	0.971	1.00e+00	1.005	1.00e+00	1.976	2.88e-05
Smc3	1.124	3.04e-01	0.939	1.00e+00	2.063	3.46e-07
Snrnp70	0.762	1.00e+00	1.03	1.00e+00	1.791	6.49e-06
Tcp1	1.147	1.85e-01	0.661	1.00e+00	1.809	3.77e-06
Tom1l2	1.079	9.36e-01	0.377	1.00e+00	1.456	3.31e-03
Tpst1	1.144	8.97e-01	1.246	5.44e-01	2.39	4.62e-05
Usp9x	0.746	1.00e+00	0.48	1.00e+00	1.226	4.96e-02

3. DOWN proteins

3.1. Common shared proteins — (all 3 contrasts)

n = 1 protein | Criteria: FDR < 0.05, |log2FC| > 1 (fold change > 2×).

Protein	MV-7 vs MV-14		MV-14 vs MV-21		MV-7 vs MV-21	
	log2FC	FDR	log2FC	FDR	log2FC	FDR
Csgalnact1	-1.844	2.38e-05	-1.334	2.22e-02	-3.178	3.37e-08

3.2. MV-7 *vs* MV-14

Exclusive proteins

n = 86 proteins | Criteria: FDR < 0.05, |log2FC| > 1 (fold change > 2×). Colored cells indicate statistically significant values, and gray cells represent not significant findings.

Protein	MV-7 *vs* MV-14		MV-14 *vs* MV-21		MV-7 *vs* MV-21	
	log2FC	FDR	log2FC	FDR	log2FC	FDR
Adamts16	-3.05	4.44e-08	2.719	9.60e-08	-0.331	1.00e+00
Adamts5	-3.576	1.51e-07	3.252	2.68e-07	-0.324	1.00e+00

Protein	MV-7 *vs* MV-14		MV-14 *vs* MV-21		MV-7 *vs* MV-21	
	log2FC	FDR	log2FC	FDR	log2FC	FDR
Ahnak2	-1.955	5.68e-03	1.259	8.24e-01	-0.696	1.00e+00
Ap2s1	-1.974	1.42e-04	1.823	6.15e-04	-0.151	1.00e+00
Atp1b1	-2.025	6.59e-06	1.88	2.93e-05	-0.145	1.00e+00
Basp1	-2.683	3.06e-06	1.77	1.82e-03	-0.914	1.00e+00
Bdh2	-1.508	1.01e-03	1.45	2.74e-03	-0.059	1.00e+00
Bop1	-1.515	1.29e-02	0.739	1.00e+00	-0.776	1.00e+00
C4b	-1.488	8.76e-04	0.817	1.00e+00	-0.672	1.00e+00
Cemip	-1.29	1.38e-02	2.006	1.41e-06	0.716	1.00e+00
Cgas	-1.429	2.05e-02	1.886	1.72e-04	0.457	1.00e+00
Clec2d	-1.547	2.12e-04	0.988	1.00e+00	-0.559	1.00e+00
Col6a5	-2.091	1.46e-06	2.35	3.32e-07	0.259	1.00e+00
Col6a6	-3.223	2.95e-07	2.788	1.41e-06	-0.435	1.00e+00
Cpne2	-1.3	9.32e-03	0.669	1.00e+00	-0.63	1.00e+00
Cpne8	-2.884	1.51e-07	2.844	1.35e-07	-0.04	1.00e+00
Cryz	-1.378	1.78e-02	0.837	1.00e+00	-0.541	1.00e+00
Csnk2a2	-1.346	1.06e-02	0.334	1.00e+00	-1.011	1.00e+00
Cthrc1	-3.538	1.60e-06	3.967	5.43e-07	0.429	1.00e+00
Cyp2f2	-1.422	1.64e-03	1.258	4.05e-02	-0.164	1.00e+00
Dhcr24	-1.425	7.90e-03	1.53	2.17e-03	0.106	1.00e+00
Dnpep	-1.375	9.28e-03	0.906	1.00e+00	-0.47	1.00e+00
Dpep1	-2.541	7.99e-06	1.234	5.00e-01	-1.307	2.16e-01
Edil3	-1.701	4.26e-05	0.589	1.00e+00	-1.112	5.97e-01
Efemp1	-3.009	1.57e-05	3.092	1.49e-05	0.083	1.00e+00
Eif2b5	-1.435	3.72e-04	1.529	9.73e-05	0.094	1.00e+00
Enpp3	-2.799	1.24e-06	2.656	2.88e-06	-0.143	1.00e+00
Fam3c	-1.279	4.28e-02	0.325	1.00e+00	-0.954	1.00e+00
Ftl1	-3.144	1.51e-07	2.674	7.14e-07	-0.47	1.00e+00
Gar1	-1.298	4.88e-02	0.533	1.00e+00	-0.765	1.00e+00
Gm49394	-3.332	7.74e-07	3.257	1.12e-06	-0.075	1.00e+00
Gnptab	-1.873	3.51e-03	1.27	5.76e-01	-0.603	1.00e+00
Gpi	-1.597	1.55e-04	0.464	1.00e+00	-1.133	4.25e-01
H2ac15	-2.405	4.41e-05	3.624	3.35e-07	1.219	6.24e-01
H2ax	-3.186	7.05e-04	4.408	2.72e-05	1.222	1.00e+00
H2az1	-2.879	7.21e-03	4.752	5.34e-05	1.873	2.55e-01
H2bc1	-3.412	6.10e-07	4.811	4.13e-08	1.399	1.31e-01
H2bc14	-3.604	6.42e-06	5.477	1.35e-07	1.873	2.08e-02
H3c11	-2.855	3.22e-04	4.547	2.17e-06	1.691	1.23e-01
H4c16	-3.128	2.38e-05	4.276	9.23e-07	1.148	1.00e+00
Hist2h2aa2	-3.105	1.93e-05	4.587	3.35e-07	1.481	1.99e-01
Hspd1	-1.748	2.52e-04	2.479	1.23e-06	0.731	1.00e+00
Ifi202	-3.031	1.51e-07	2.97	1.21e-07	-0.06	1.00e+00
Igf2	-4.077	1.82e-06	3.169	3.87e-05	-0.907	1.00e+00
Igsf10	-1.754	8.68e-06	2.471	8.59e-08	0.718	1.00e+00
Igsf8	-2.016	1.93e-05	1.848	1.00e-04	-0.168	1.00e+00
Il1rap	-1.844	1.00e-05	1.512	6.61e-04	-0.332	1.00e+00
Kdsr	-1.379	2.17e-02	1.338	4.73e-02	-0.04	1.00e+00
Ldhb	-3.314	4.62e-07	2.682	6.13e-06	-0.632	1.00e+00
Lix1l	-1.661	1.94e-04	2.091	4.13e-06	0.431	1.00e+00
Lman2	-1.255	4.97e-02	0.096	1.00e+00	-1.159	3.08e-01

Protein	MV-7 *vs* MV-14		MV-14 *vs* MV-21		MV-7 *vs* MV-21	
	log2FC	FDR	log2FC	FDR	log2FC	FDR
Lox	-1.245	3.71e-02	0.894	1.00e+00	-0.35	1.00e+00
Loxl1	-2.484	3.51e-07	2.891	8.59e-08	0.407	1.00e+00
Macroh2a2	-2.136	1.46e-06	2.421	3.13e-07	0.285	1.00e+00
Man2a1	-1.495	6.07e-03	0.658	1.00e+00	-0.837	1.00e+00
Marcksl1	-2.938	7.08e-05	2.443	8.48e-04	-0.495	1.00e+00
Mesd	-2.049	9.37e-07	1.105	6.47e-01	-0.944	1.00e+00
Mfge8	-2.433	4.49e-07	1.476	2.49e-03	-0.957	1.00e+00
Naalad2	-2.055	6.45e-06	1.86	4.16e-05	-0.195	1.00e+00
Naglu	-1.316	1.08e-02	0.386	1.00e+00	-0.93	1.00e+00
Ncl	-1.985	1.68e-05	1.547	1.98e-03	-0.439	1.00e+00
Npc2	-1.294	1.34e-02	0.11	1.00e+00	-1.183	1.34e-01
Npm1	-2.279	6.56e-07	1.348	1.45e-02	-0.931	1.00e+00
Nrdc	-2.416	2.91e-07	2.207	8.75e-07	-0.209	1.00e+00
Nsdhl	-1.767	2.83e-03	1.704	5.81e-03	-0.062	1.00e+00
Nucb1	-1.99	1.02e-04	0.893	1.00e+00	-1.096	1.00e+00
Pepd	-1.46	1.18e-02	0.81	1.00e+00	-0.65	1.00e+00
Plpp3	-1.397	4.56e-03	0.328	1.00e+00	-1.069	1.00e+00
Polr2a	-2.085	8.55e-06	2.035	1.62e-05	-0.051	1.00e+00
Polr2b	-1.995	4.50e-06	2.063	3.28e-06	0.068	1.00e+00
Polr2c	-2.107	2.53e-05	1.674	1.50e-03	-0.433	1.00e+00
Polr2i	-1.48	2.93e-04	1.822	4.85e-06	0.342	1.00e+00
Psap	-1.414	4.98e-03	0.796	1.00e+00	-0.618	1.00e+00
Psmb5	-1.948	2.97e-06	1.76	2.43e-05	-0.187	1.00e+00
Ptma	-1.729	1.67e-04	1.614	7.54e-04	-0.115	1.00e+00
Rab9a	-1.888	1.68e-06	0.863	1.00e+00	-1.024	1.00e+00
Rbp1	-1.645	4.00e-04	2.072	8.75e-06	0.427	1.00e+00
Sdf4	-1.829	1.01e-03	0.721	1.00e+00	-1.109	1.00e+00
Slc16a1	-1.276	1.74e-02	0.355	1.00e+00	-0.92	1.00e+00
Slit3	-3.258	1.97e-08	3.032	4.13e-08	-0.227	1.00e+00
Smpd2	-1.338	1.18e-02	0.618	1.00e+00	-0.72	1.00e+00
Steap3	-2.447	3.51e-07	2.262	9.53e-07	-0.186	1.00e+00
Tmed5	-2.562	8.72e-05	1.282	6.29e-01	-1.28	5.68e-01
Tnxb	-2.59	1.24e-06	2.185	1.62e-05	-0.405	1.00e+00
Utp18	-2.003	5.62e-05	1.955	9.93e-05	-0.048	1.00e+00
Vcan	-1.768	2.99e-05	1.859	1.57e-05	0.091	1.00e+00

Shared proteins compared with MV-7 *vs* MV-21

n = 18 proteins | Criteria: FDR < 0.05, |log2FC| > 1 (fold change > 2×). Colored cells indicate statistically significant values, and gray cells represent not significant findings.

Protein	MV-7 vs MV-14		MV-14 vs MV-21		MV-7 vs MV-21	
	log2FC	FDR	log2FC	FDR	log2FC	FDR
Anpep	-1.534	9.00e-04	-0.194	1.00e+00	-1.728	9.34e-05
Galnt1	-1.291	2.93e-02	-0.241	1.00e+00	-1.532	7.40e-04
Galnt7	-1.323	2.17e-02	-0.118	1.00e+00	-1.441	3.76e-03
Gng12	-1.34	3.56e-03	-0.112	1.00e+00	-1.452	5.31e-04
Gpnmb	-2.705	5.48e-07	0.628	1.00e+00	-2.077	2.29e-05
Lman1	-1.471	2.44e-03	0.126	1.00e+00	-1.345	1.84e-02
Mix23	-1.492	1.45e-03	-0.037	1.00e+00	-1.529	9.52e-04
Polr3a	-3.484	2.88e-07	1.244	4.55e-01	-2.24	5.50e-05
Pvr	-3.248	1.51e-07	1.65	1.82e-03	-1.598	3.07e-03
Set	-3.168	3.06e-07	1.499	1.80e-02	-1.669	2.67e-03
Slc2a1	-2.08	1.08e-06	0.478	1.00e+00	-1.602	1.35e-04
Thy1	-2.744	2.21e-06	0.984	1.00e+00	-1.76	1.73e-03
Tmed10	-1.395	1.24e-02	-0.766	1.00e+00	-2.162	4.42e-06
Tmed4	-1.706	2.46e-04	0.049	1.00e+00	-1.657	4.93e-04
Tmed7	-1.555	6.26e-04	-0.566	1.00e+00	-2.122	2.32e-06
Tmed9	-1.425	1.45e-03	-0.168	1.00e+00	-1.593	1.36e-04
Wfs1	-2.047	4.55e-03	0.134	1.00e+00	-1.914	1.18e-02
Xpnpep2	-2.264	2.72e-06	0.943	1.00e+00	-1.321	4.94e-02

3.3. MV-14 vs MV-21

Exclusive proteins

n = 70 proteins | Criteria: FDR < 0.05, |log2FC| > 1 (fold change > 2×). Colored cells indicate statistically significant values, and gray cells represent not significant findings.

Protein	MV-7 *vs* MV-14		MV-14 *vs* MV-21		MV-7 *vs* MV-21	
	log2FC	FDR	log2FC	FDR	log2FC	FDR
Abca8b	0.827	1.00e+00	-1.586	6.92e-04	-0.759	1.00e+00
Abcc1	0.598	1.00e+00	-1.363	2.21e-02	-0.765	1.00e+00
Ak1	2.224	1.13e-05	-1.601	3.22e-03	0.623	1.00e+00
Anxa6	1.42	1.01e-03	-1.542	1.69e-04	-0.121	1.00e+00
Anxa8	2.565	2.97e-06	-2.083	7.36e-05	0.482	1.00e+00
Ass1	1.276	3.66e-02	-1.328	1.80e-02	-0.052	1.00e+00
C3	1.138	4.56e-01	-1.523	1.47e-03	-0.385	1.00e+00
Calhm5	0.51	1.00e+00	-1.507	4.95e-04	-0.996	1.00e+00
Calm1	0.81	1.00e+00	-1.651	2.38e-03	-0.842	1.00e+00
Cav1	0.538	1.00e+00	-1.472	1.57e-03	-0.934	1.00e+00
Cd44	1.181	3.10e-01	-1.442	8.37e-03	-0.261	1.00e+00
Clic4	0.96	1.00e+00	-1.403	1.19e-02	-0.443	1.00e+00
Cnp	1.831	4.46e-06	-1.414	1.42e-03	0.417	1.00e+00
Col12a1	1.56	1.58e-03	-1.556	1.95e-03	0.004	1.00e+00
Dcxr	1.015	1.00e+00	-1.449	3.11e-03	-0.434	1.00e+00
Dstn	0.812	1.00e+00	-1.509	1.32e-02	-0.698	1.00e+00
Ece1	0.846	1.00e+00	-1.342	1.53e-02	-0.496	1.00e+00
Ehd1	0.917	1.00e+00	-1.372	6.04e-03	-0.454	1.00e+00
Ephx1	1.095	1.00e+00	-2.245	1.21e-04	-1.15	1.00e+00

Protein	MV-7 *vs* MV-14		MV-14 *vs* MV-21		MV-7 *vs* MV-21	
	log2FC	FDR	log2FC	FDR	log2FC	FDR
F13a1	0.613	1.00e+00	-1.521	5.48e-04	-0.908	1.00e+00
Fabp5	0.811	1.00e+00	-1.497	5.04e-04	-0.686	1.00e+00
Fat1	2.098	2.42e-06	-1.354	1.45e-02	0.745	1.00e+00
Fbn2	2.722	2.95e-07	-2.86	1.70e-07	-0.138	1.00e+00
Gpx1	0.85	1.00e+00	-1.449	4.42e-02	-0.598	1.00e+00
Gsdmd	1.692	7.19e-05	-2.118	1.77e-06	-0.426	1.00e+00
H1-0	2.986	1.97e-08	-2.289	2.07e-07	0.697	1.00e+00
Hnrnpf	0.897	1.00e+00	-1.545	2.66e-03	-0.648	1.00e+00
Htra1	2.319	3.97e-07	-1.262	4.89e-02	1.057	1.00e+00
IGK	1.207	2.74e-01	-2.366	2.88e-06	-1.159	5.56e-01
Igfbp4	1.115	4.87e-01	-1.591	1.83e-04	-0.476	1.00e+00
Inf2	0.44	1.00e+00	-1.347	2.25e-02	-0.907	1.00e+00
Ly75	1.735	1.02e-04	-2.192	2.38e-06	-0.457	1.00e+00
Mdh2	0.776	1.00e+00	-1.703	2.78e-05	-0.927	1.00e+00
Mme	0.971	1.00e+00	-2.002	9.80e-07	-1.031	1.00e+00
Mrps26	1.813	3.15e-03	-1.67	1.30e-02	0.143	1.00e+00
Mtpn	1.086	8.54e-01	-1.314	3.05e-02	-0.228	1.00e+00
Niban1	1.809	1.43e-05	-1.862	1.09e-05	-0.053	1.00e+00
Npepl1	0.953	1.00e+00	-1.376	1.53e-02	-0.423	1.00e+00
P10404	2.163	4.48e-05	-2.553	5.41e-06	-0.39	1.00e+00
P2rx7	0.883	1.00e+00	-1.726	2.66e-05	-0.843	1.00e+00
P2ry10b	1.468	4.44e-03	-1.872	5.10e-05	-0.404	1.00e+00
Pde8a	2.466	2.88e-07	-2.187	1.12e-06	0.279	1.00e+00
Pdia4	0.439	1.00e+00	-1.3	4.97e-02	-0.861	1.00e+00
Pip4p2	1.585	2.46e-04	-1.73	5.10e-05	-0.145	1.00e+00
Pltp	2.024	3.28e-05	-1.794	3.05e-04	0.23	1.00e+00
Prkaa1	0.565	1.00e+00	-1.584	2.79e-04	-1.019	1.00e+00
Psmb8	2.414	4.00e-04	-3.077	2.47e-05	-0.663	1.00e+00
Ptpra	0.61	1.00e+00	-1.541	2.83e-04	-0.931	1.00e+00
Ptx3	0.335	1.00e+00	-1.36	6.06e-03	-1.026	1.00e+00
Rab21	1.383	3.58e-02	-1.657	1.62e-03	-0.274	1.00e+00
Rap1b	0.901	1.00e+00	-1.535	1.77e-03	-0.633	1.00e+00
S100a10	1.877	2.55e-05	-1.788	7.43e-05	0.089	1.00e+00
S100a13	2.226	3.08e-06	-2.669	3.35e-07	-0.443	1.00e+00
S1pr1	1.393	1.18e-02	-1.675	3.36e-04	-0.281	1.00e+00
Scrn1	2.219	1.08e-06	-1.72	8.59e-05	0.499	1.00e+00
Serpinb6	0.369	1.00e+00	-1.259	4.33e-02	-0.891	1.00e+00
Serpine2	2.063	1.24e-06	-1.415	2.31e-03	0.647	1.00e+00
Sgcb	2.407	9.31e-04	-3.241	2.93e-05	-0.835	1.00e+00
Sgcd	1.206	3.21e-01	-1.976	7.34e-05	-0.771	1.00e+00
Slpi	1.065	9.35e-01	-1.419	1.52e-03	-0.355	1.00e+00
Smpdl3b	0.597	1.00e+00	-1.745	8.97e-04	-1.147	7.52e-01
Sord	1.46	9.69e-04	-1.684	5.16e-05	-0.224	1.00e+00
Tagln2	0.959	1.00e+00	-1.352	1.59e-02	-0.393	1.00e+00
Thbs1	2.375	1.13e-06	-1.762	1.55e-04	0.614	1.00e+00
Thsd4	3.074	6.26e-07	-1.936	4.07e-04	1.139	9.59e-01
Tmem43	0.54	1.00e+00	-1.38	2.63e-02	-0.841	1.00e+00
Tnfrsf10b	1.489	3.71e-02	-1.809	2.21e-03	-0.321	1.00e+00
Tpm1	1.95	3.23e-05	-1.376	2.76e-02	0.574	1.00e+00

Protein	MV-7 *vs* MV-14		MV-14 *vs* MV-21		MV-7 *vs* MV-21	
	log2FC	FDR	log2FC	FDR	log2FC	FDR
Vcam1	0.904	1.00e+00	-1.321	7.09e-03	-0.417	1.00e+00
Vim	1.502	2.72e-03	-1.864	5.10e-05	-0.362	1.00e+00

Shared proteins compared with MV-7 *vs* MV-21

n = 15 proteins | Criteria: FDR < 0.05, |log2FC| > 1 (fold change > 2×). Colored cells indicate statistically significant values, and gray cells represent not significant findings.

Protein	MV-7 vs MV-14		MV-14 vs MV-21		MV-7 vs MV-21	
	log2FC	FDR	log2FC	FDR	log2FC	FDR
Acp3	0.18	1.00e+00	-3.621	6.26e-08	-3.44	5.93e-08
Bst2	0.077	1.00e+00	-1.762	6.92e-06	-1.686	1.50e-05
Cd200	1.071	1.00e+00	-2.576	4.54e-07	-1.505	3.24e-03
Enpep	0.13	1.00e+00	-1.542	1.41e-03	-1.411	8.01e-03
Fas	0.913	1.00e+00	-2.607	8.59e-08	-1.693	4.09e-05
Hp	0.923	1.00e+00	-2.253	2.11e-07	-1.33	5.26e-03
Ifit1	-0.453	1.00e+00	-2.204	5.81e-06	-2.657	2.96e-07
Nt5e	0.446	1.00e+00	-3.065	4.13e-08	-2.62	7.96e-08
Pdpn	0.946	1.00e+00	-3.322	8.73e-09	-2.376	9.18e-08
Raet1e	0.686	1.00e+00	-2.601	8.75e-06	-1.915	7.08e-04
Slc39a14	0.782	1.00e+00	-2.666	2.58e-07	-1.885	3.70e-05
Sod2	-0.026	1.00e+00	-2.376	1.07e-07	-2.402	6.09e-08
Sphk1	0.318	1.00e+00	-2.042	2.99e-05	-1.723	5.07e-04
Taldo1	-0.014	1.00e+00	-1.213	4.13e-02	-1.227	2.70e-02
Tgm2	0.827	1.00e+00	-2.447	2.11e-07	-1.621	1.61e-04

3.4. MV-7 *vs* MV-21

Exclusive proteins

n = 25 proteins | Criteria: FDR < 0.05, |log2FC| > 1 (fold change > 2×). Colored cells indicate statistically significant values, and gray cells represent not significant findings.

Protein	MV-7 *vs* MV-14		MV-14 *vs* MV-21		MV-7 *vs* MV-21	
	log2FC	FDR	log2FC	FDR	log2FC	FDR
Arsb	-0.621	1.00e+00	-0.688	1.00e+00	-1.309	2.76e-02
Atl3	-0.985	1.00e+00	-0.417	1.00e+00	-1.402	1.36e-02
Atp6v1d	-0.924	1.00e+00	-0.453	1.00e+00	-1.376	2.11e-02
Bri3bp	-0.32	1.00e+00	-1.119	1.00e+00	-1.44	2.69e-02
Calr	-1.117	5.33e-01	-0.686	1.00e+00	-1.803	2.62e-05
Canx	-0.233	1.00e+00	-1.023	1.00e+00	-1.256	4.69e-02
Cd47	-0.149	1.00e+00	-1.327	1.05e-01	-1.476	1.51e-02
Cp	-0.732	1.00e+00	-0.691	1.00e+00	-1.422	5.34e-03
Cxcl12	-0.733	1.00e+00	-1.149	3.53e-01	-1.882	6.49e-06
Cyb5b	-0.34	1.00e+00	-0.977	1.00e+00	-1.317	1.10e-02
Ddah2	-0.398	1.00e+00	-0.852	1.00e+00	-1.249	4.39e-02
Gns	-1.171	1.57e-01	-0.123	1.00e+00	-1.294	1.51e-02
Ifi204	-0.67	1.00e+00	-1.104	7.58e-01	-1.774	2.22e-05
Ifitm3	-0.999	1.00e+00	-1.034	1.00e+00	-2.033	5.21e-04
Lgals3	-0.575	1.00e+00	-0.75	1.00e+00	-1.325	2.43e-02
Lta4h	-0.163	1.00e+00	-1.147	5.15e-01	-1.31	3.32e-02

**Protein**	**MV-7 *vs* MV-14**		**MV-14 *vs* MV-21**		**MV-7 *vs* MV-21**	
	**log2FC**	**FDR**	**log2FC**	**FDR**	**log2FC**	**FDR**
Manf	-0.546	1.00e+00	-0.932	1.00e+00	-1.478	1.03e-03
Mfap5	-0.693	1.00e+00	-0.687	1.00e+00	-1.38	1.49e-02
Plbd2	-0.519	1.00e+00	-0.869	1.00e+00	-1.387	4.70e-03
Rtn3	-0.944	1.00e+00	-0.859	1.00e+00	-1.802	8.93e-05
Rtn4	-1.514	3.24e-01	-1.151	1.00e+00	-2.666	1.37e-03
Serinc1	-0.97	1.00e+00	-0.695	1.00e+00	-1.665	8.44e-05
Tmed3	-0.75	1.00e+00	-0.532	1.00e+00	-1.282	3.96e-02
Tmem87b	-0.96	1.00e+00	-0.529	1.00e+00	-1.489	8.29e-04
VTN	-0.818	1.00e+00	-0.489	1.00e+00	-1.307	1.65e-02

Supporting Information 2

Gene ontology (GO) enrichment analysis on the sets of unique proteins identified across pairwise temporal contrasts (MV-7, MV-14, MV-21).

Matrix vesicles (MV); biological process (BP); cellular compartment (CC); molecular function (MF).

1. MV-7 *vs* MV-14

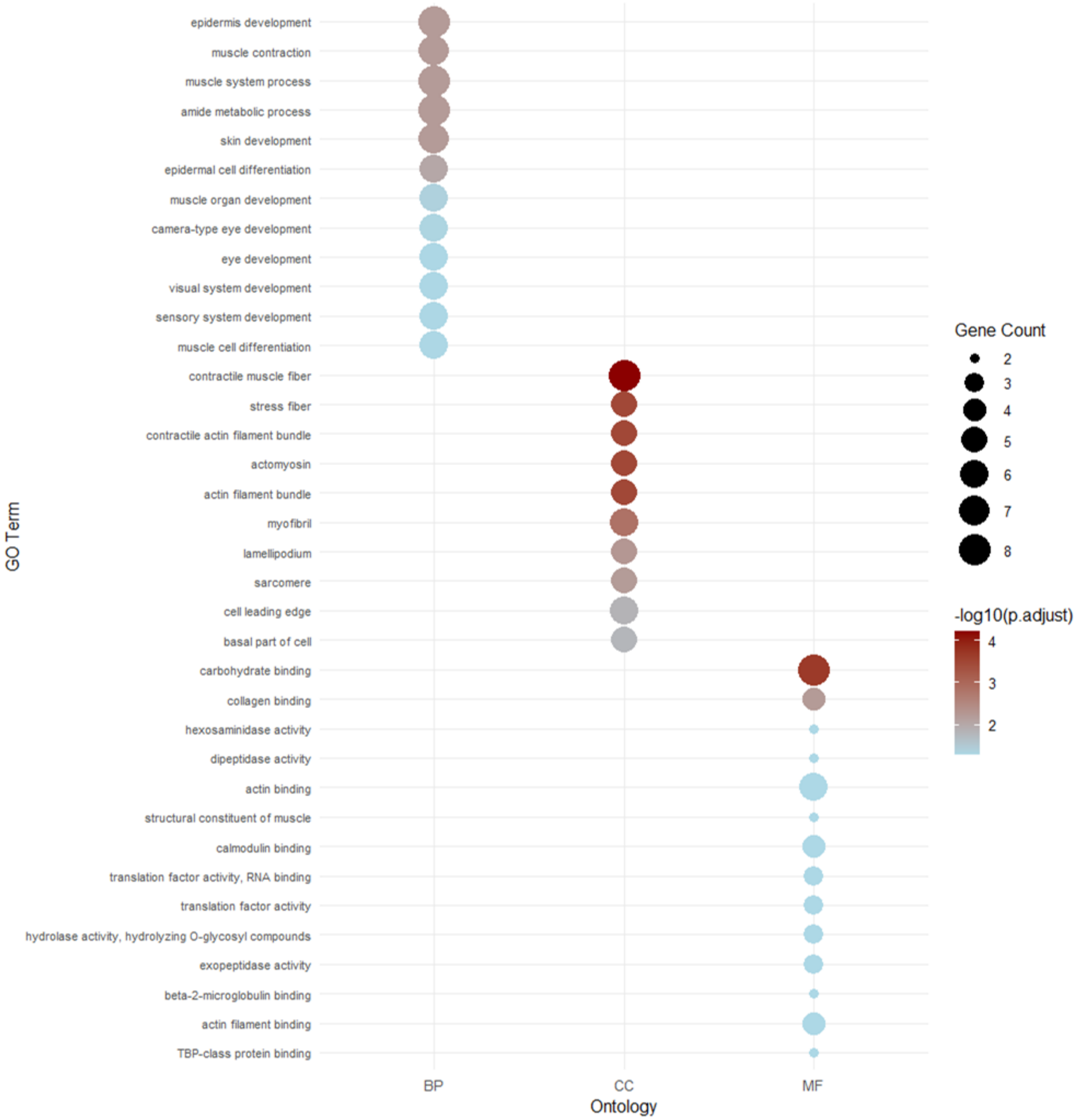

2. MV-14 *vs* MV-21

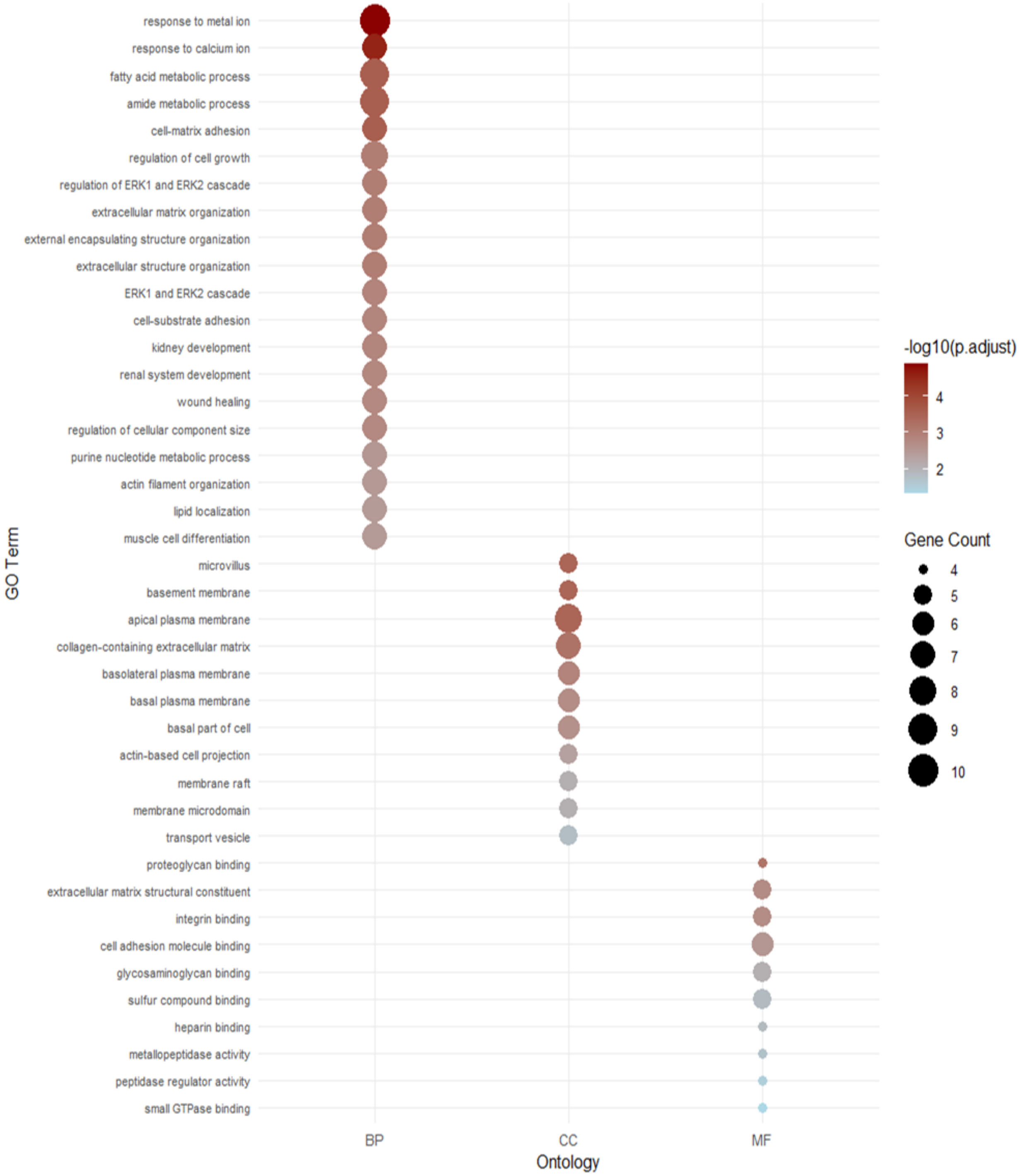

3. MV-7 *vs* MV-21

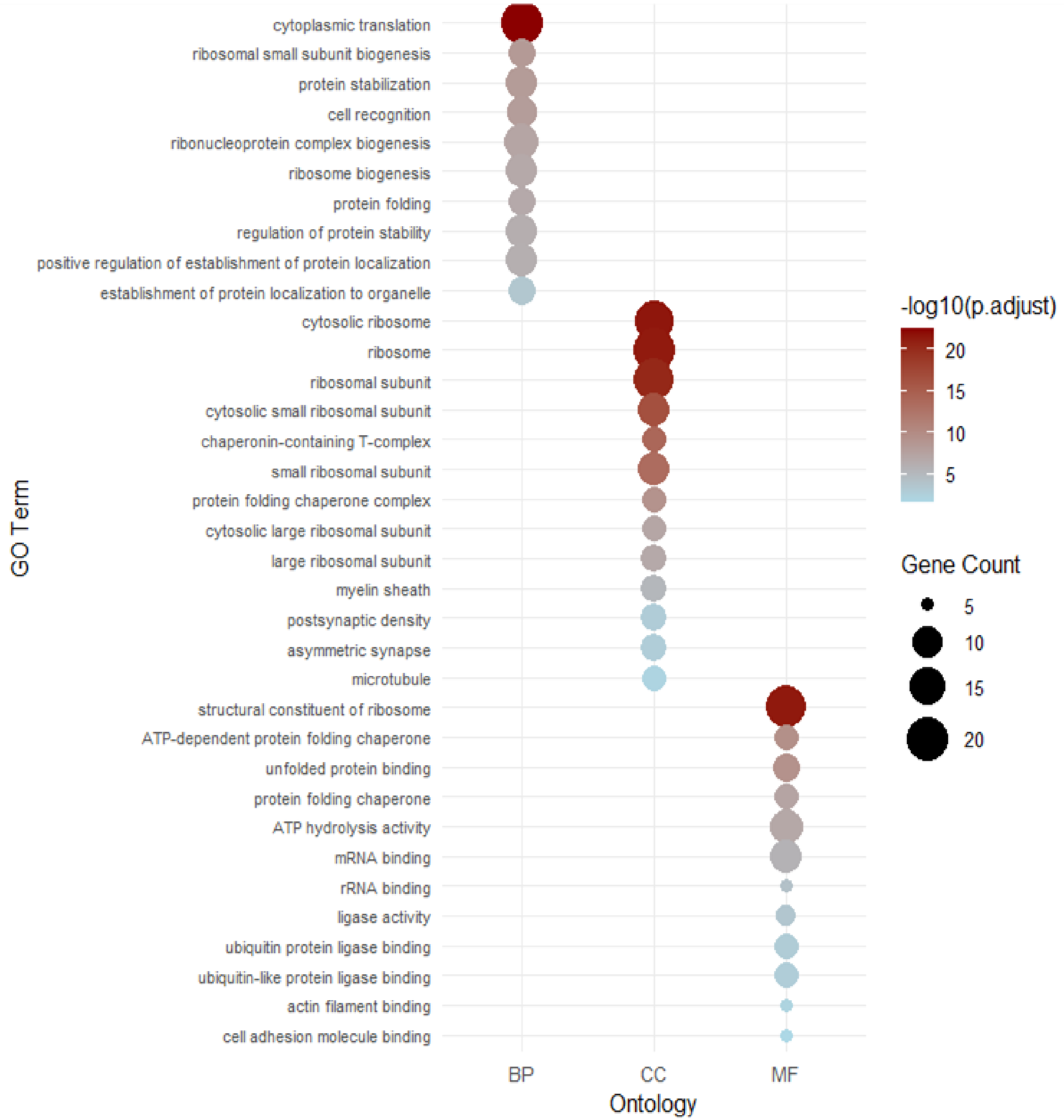

cytoplasmic translation
ribosomal small subunit biogenesis
protein stabilization
cell recognition
ribonucleoprotein complex biogenesis
ribosome biogenesis
protein folding
regulation of protein stability
positive regulation of establishment of protein localization
establishment of protein localization to organelle
cytosolic ribosome
ribosome
ribosomal subunit
cytosolic small ribosomal subunit
chaperonin-containing T-complex
small ribosomal subunit
protein folding chaperone complex
cytosolic large ribosomal subunit
large ribosomal subunit
myelin sheath
postsynaptic density
asymmetric synapse
microtubule
structural constituent of ribosome
ATP-dependent protein folding chaperone
unfolded protein binding
protein folding chaperone
ATP hydrolysis activity
mRNA binding
rRNA binding
ligase activity
ubiquitin protein ligase binding
ubiquitin-like protein ligase binding
actin filament binding
cell adhesion molecule binding
GO Term
BP
CC
MF
Ontology
-log10(p.adjust)
20
15
10
5
Gene Count
5
10
15
20
